# Rapid Deep Vat Printing Using Photoclickable Collagen‐Based Bioresins

**DOI:** 10.1002/adhm.202405105

**Published:** 2025-07-04

**Authors:** Michael Winkelbauer, Amelia Hasenauer, Dominic Rütsche, Hao Liu, Jakub Janiak, Michael Nguyen, Karen L. Christman, Marcy Zenobi‐Wong, Parth Chansoria

**Affiliations:** ^1^ Department of Health Sciences and Technology ETH Zürich 8093 Switzerland; ^2^ Department of Surgery University Children's Hospital Zürich 8008 Switzerland; ^3^ Shu Chien‐Gene Lay Department of Bioengineering Sanford Stem Cell Institute Sanford Consortium for Regenerative Medicine University of California San Diego La Jolla CA 92093 USA

**Keywords:** collagen, FLight printing, photoclick, thiol‐ene, tissue interface, tomographic printing

## Abstract

Deep vat printing (DVP) techniques, including tomographic and filamented light (Flight) printing have transformed the tissue engineering landscape by enabling layer‐free bioprinting at high resolution and speeds. Importantly, collagen is one of the most widely used materials in tissue biofabrication, but DVP using collagen‐based photoresins has been under‐explored. In this work, photoclickable collagen‐based resins are demonstrated which are compatible with DVP, enabling prints with up to 50 µm print resolution and speeds less than 20 s per cm^3^. These resins contain iodixanol, which acts as a refractive index matching agent to reduce optical inhomogeneities by added cells and improves the stability of the collagen‐based resins under neutralized conditions. As a potential application, multi‐material tomographic and FLight printing is demonstrated to create intricate tissue constructs featuring muscle and connective tissue interfaces. While tomographic printing allows greater complexity over the overall shape of the constructs, FLight constructs demonstrate enhanced biomimicry through generation of aligned multinucleated myotubes, transition zones between the two cell types, and regularly spaced sarcomere structure (≈2.6 µm average sarcomere length) which facilitates contractility in the muscle regions. The demonstration of photoclickable, isotonic and neutral collagen‐based bioresins offers a new solution for DVP of biomimetic complex tissues and grafts.

## Graphical abstract

Collagen‐based resin formulations based on thiol‐ene photoclick chemistry are demonstrated. These resins enable easy handleability under neutral conditions and further allow facile fabrication of multicellular tissue constructs via tomographic or FLight printing. It is shown that while tomographic printing enables printing of complex 3D shapes, FLight printing produces 2.5D shapes with cell guiding microarchitectures for improved biomimicry relevant to aligned tissues.

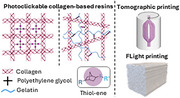

## 1 Introduction

Additive manufacturing, i.e., 3D printing, has revolutionized tissue engineering by enabling the creation of complex and patient‐specific tissue constructs. Recently developed bioprinting tools such as deep vat printing (DVP),^[^ ^1^ ^]^ offer exciting new avenues in tissue engineering as they enable the layer‐less fabrication of 3D shapes with unprecedented speed (several seconds), cell viability (> 95%), and resolution (up to 20µm).^[^ ^2^, ^3^, ^4^ ^]^ A variety of deep vat printing approaches exist, including tomographic printing,^[^ ^5^, ^6^ ^]^ multidirectional projection,^[^ ^7^, ^8^ ^]^ light sheet‐based printing (e.g., Xolography),^[^ ^9^, ^10^ ^]^ and filamented light (FLight) printing.^[^ ^11^, ^12^, ^13^ ^]^ Amongst these approaches, tomographic, light sheet‐ and multidirectional projection‐based printing create 3D shapes by achieving a cumulative light dose necessary for the photopolymerization of the resins. Notably, tomographic printing, is the most advanced and extensively studied technique of the aforementioned methods due to its applications across printing of complex tissue‐engineered constructs,^[^ ^5^, ^14^ ^]^ inorganic materials (e.g., glass),^[^ ^15^ ^]^ and prototypes.^[^ ^6^, ^16^ ^]^ This technique involves the projection of dynamically evolving images (derived through the radon transformation of a 3D object) into a photoresin‐containing vial to achieve the cumulative light dose required for material cross‐linking.^[^ ^2^, ^6^ ^]^ In contrast, FLight printing relies on the interaction of a speckled laser intensity distribution with a photocrosslinkable resin, which results in the formation of self‐focused light beams (due to a change in refractive index upon cross‐linking) within the resin.^[^ ^1^, ^11^ ^]^ Upon reaching the cumulative light dose necessary for cross‐linking, the resins preferentially cross‐link further along the self‐focused light beams, resulting in the formation of continuous microfilamented constructs.^[^ ^1^ ^]^ Similar to other DVP techniques, FLight is compatible with cellular resins and naturally‐ or synthetically‐derived biomaterials, and the microfilaments are excellent guidance cues for anisotropic cellular organization and matrix secretion. Accordingly, this technique is especially suited toward anisotropic tissue engineering (e.g., muscles,^[^ ^11^, ^17^ ^]^ nerves,^[^ ^12^ ^]^ articular cartilage,^[^ ^13^ ^]^ tendons,^[^ ^18^ ^]^ etc.).

Tomographic and FLight printing offer unique potential for fundamental and translational research, and the used materials are constantly evolving to meet the needs of the tissue engineering field. While previously developed materials for tomographic and FLight printing primarily focused on chain growth polymerization (i.e., acrylate‐ or methacrylate‐based polymers), photoclickable materials based on step‐growth cross‐linking have brought forth a paradigm shift, particularly with regard to biomedical applications.^[^ ^13^, ^19^, ^20^ ^]^ Photoclick reactions are versatile, highly efficient, and selective processes that operate under mild conditions, producing non‐toxic byproducts.^[^ ^21^ ^]^ Among these, the thiol‐ene reaction is the most commonly used,^[^ ^22^, ^23^ ^]^ where photoinitiator‐generated radicals abstract hydrogen from thiol groups, initiating the formation of thiyl radicals. The thiyl radical then reacts with the double bond of an alkene, creating a carbon‐centered radical that subsequently abstracts hydrogen from another thiol, generating a new thiyl radical. Compared to chain growth polymerization, a step growth polymerization rapidly proceeds and is insensitive to oxygen inhibition. Additionally, step‐growth mechanisms result in more uniform and predictable networks, with reduced shrinkage and improved mechanical properties (e.g., higher toughness).^[^ ^22^, ^23^ ^]^

Despite significant progress in deep vat printing (DVP) techniques, the development of collagen and collagen‐based resin formulations has been limited. This is because the resins based on collagen typically suffer from poor print fidelity and handling limitations, despite offering improved bioactivity. Here, the development of a photoclickable collagen‐based resin, which enables biochemical relevance with mechanical tunability and high‐resolution printability using DVP is of high relevance and importance to the biomedical community. Collagen is the most abundant protein in the extracellular matrix of various tissues and makes up ≈30% of the total protein mass in humans.^[^ ^24^ ^]^ In its fibrillar form it provides strength and elasticity to the tissues, and plays a critical role in biological processes including cell adhesion, tissue regeneration, wound healing, and cell‐cell signaling through long range mechanotransduction enabled by the collagen fibers.^[^ ^25^, ^26^ ^]^ In gelatin‐based biomaterials, the major cell binding motif is the RGD sequence, which enables cell attachment via integrins α5β1 and αvβ3.^[^ ^27^ ^]^ In contrast, in collagen‐1, the RGD moieties are constrained within the triple helix, and the cell‐binding ligands consist of GxOGER sequences (G is glycine, O is hydroxyproline, E is glutamate, R is arginine, and x is a hydrophobic amino acid such as phenylalanine). Cells engage with these ligands via integrins α1β1, α2β1, α10β1, and α11β1.^[^ ^28^, ^29^, ^30^ ^]^ Consequently, the transformation of collagen into gelatin shifts the binding specificity from α1β1, α2β1, α10β1, and α11β1 integrins to α5β1 and αVβ3 integrins,^[^ ^28^ ^]^ which can lead to significant alterations in signaling pathways linked to tissue homeostasis, differentiation, and cell migration.^[^ ^31^ ^]^ Cumulating on these findings, collagen‐based resins more closely resemble the extracellular matrix, providing a more physiologically relevant environment for enhancing cell attachment, proliferation, and differentiation.^[^ ^27^, ^29^, ^30^ ^]^ These attributes make collagen‐based resins particularly advantageous for creating functional tissue constructs that more accurately reflect the in vivo matrix environment in biomedical applications. Notably, photoclickable decellularized extracellular (dECM) materials have been used for both tomographic,^[^ ^32^ ^]^ and FLight printing.^[^ ^33^ ^]^ These approaches rely on the use of ruthenium/sodium persulfate‐based photoinitiation for cross‐linking the native tyrosine groups in the resins, which enables a modification‐free approach for rapid photo‐crosslinking. However, the resulting constructs are soft and prone to rapid degradation in vitro and in vivo and require either the use of additional supporting material, or high starting material concentration, which increases complexity and costs.^[^ ^32^ ^]^ Further, the dECM resins present several challenges including their variable biochemical composition of proteins and biomolecules due to biological variability, potential retention of immunogenic components like residual DNA, or potential batch‐to‐batch variability during production, which can impair reproducibility and consistency.^[^ ^34^ ^]^ Accordingly, a collagen‐based resin formulation with well‐defined constitutive components and utilizing thiol‐ene photoclick chemistry allows the formation of robust biomaterial constructs with improved reproducibility and translational potential.

**Figure 1:**
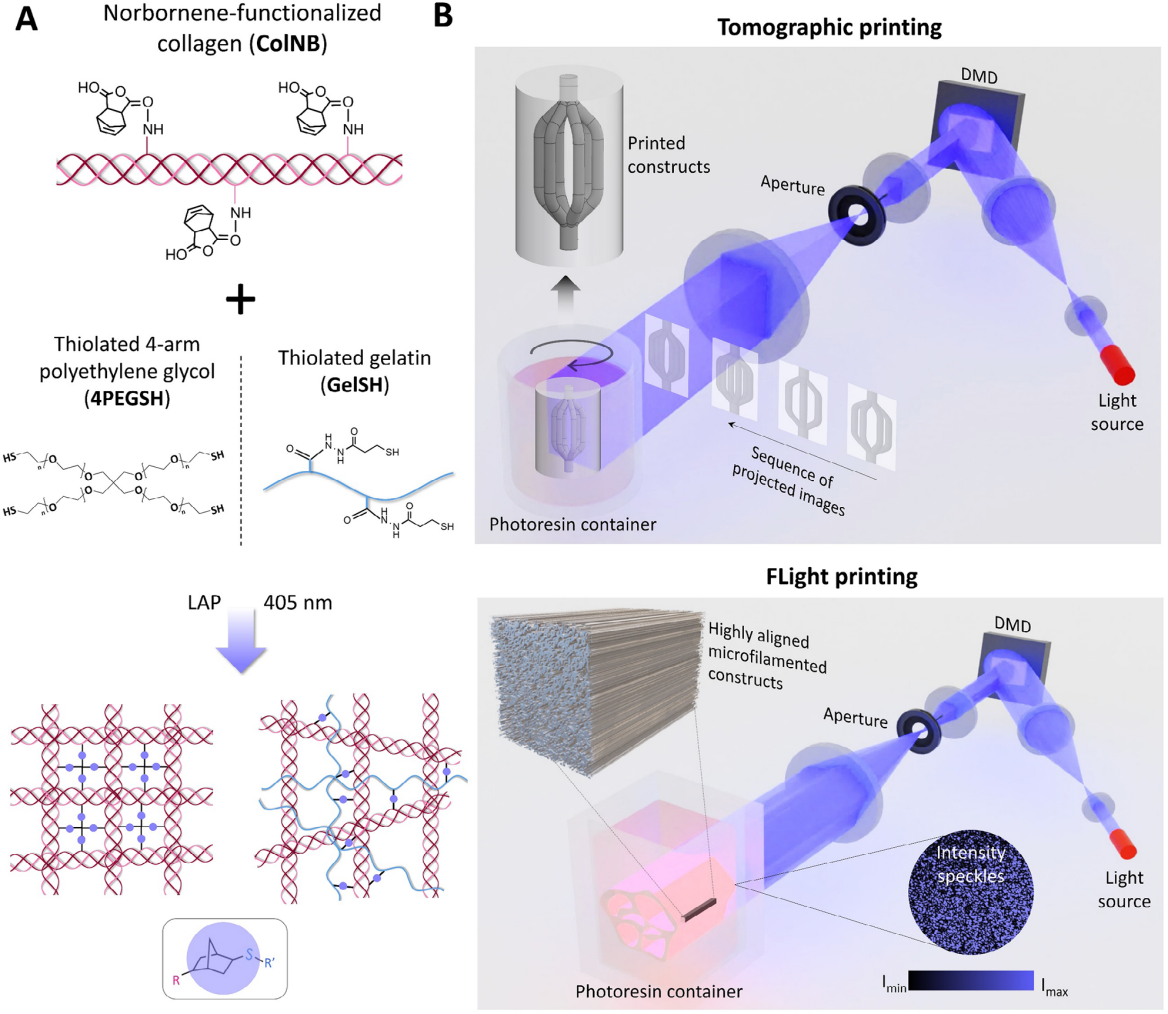
Photoclickable collagen‐based resins and fabrication methods used in the study. A) Collagen formulations were norbornene‐functionalized and mixed with thiolated crosslinkers (4‐arm thiolated PEG, or thiolated gelatin). Exposure of the resins to controlled light doses at 405 nm allowed fabrication of complex biomaterial scaffolds. B) We used the photoclickable collagen‐based resins in two different techniques – tomographic printing and FLight printing – and evaluated the characteristics of the fabricated constructs.

In this work, we demonstrate collagen‐based photoresins (**Figure** **1**A) using norbornene‐functionalized collagen in combination with two different types of thiolated crosslinkers: 1) 4‐arm thiolated polyethylene glycol (4PEGSH), or 2) Thiolated gelatin (GelSH). We utilize these resins in both tomographic and FLight printing approaches (Figure 1B), to be able to compare the two techniques in terms of the complexity of shapes one can produce, and the microarchitectural characteristics one can achieve. Due to the unique attributes of each approach, we highlight differences in the characteristics of the bioprinted tissues upon maturation. Finally, we discuss future perspectives which may guide researchers in using the optimal processing approach for their applications, enabling the development of new collagen‐based resins which can improve the biomimicry of engineered tissues and grafts.

## 2 Results

**Figure 2:**
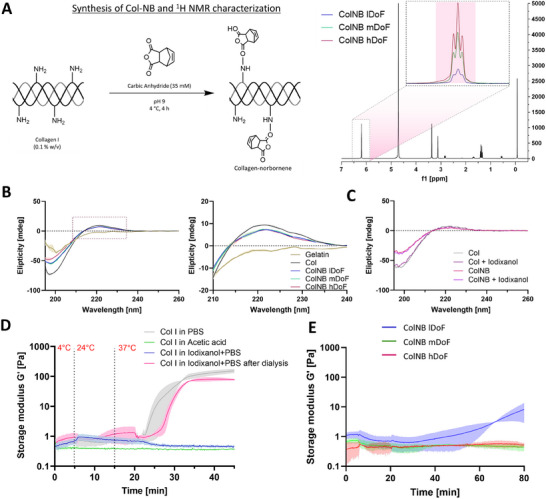
ColNB synthesis and characterization. A) Synthesis of norbornene‐functionalized collagen and ^1^H NMR curve (right) demonstrating the signal at 6–6.2 ppm ascribed to the alkene peaks in the norbornene group.^[^ ^42^ ^]^ The integrals of the peaks in the three ColNB groups (low, medium or high degree of functionalization (lDoF, mDoF and hDoF, respectively)) were compared to the methyl protons of the DSS internal standard (−0.05 to 0.05 ppm).^[^ ^36^, ^37^ ^]^ B) Circular dichroism (CD) measurements of collagen, ColNB and gelatin. A slight reduction of the characteristic positive peak at 220 nm and negative peak at 198 nm (conforming to the triple helix structure of collagen) after norbornene functionalization is consistent with existing studies.^[^ ^35^, ^36^ ^]^ CD spectra were smoothed using Savitzky–Golay filter (window length: 15, third‐order polynomial function). C) Here, the addition of iodixanol (which is removed through dialysis prior to CD measurement to prevent interference with measurements) does not affect the conformation of either pristine collagen or ColNB. D) Thermorheological responses of pristine Col I formulations. Here, the groups containing Iodixanol do not undergo physical gelation, but regain their physical gelation potential after the iodixanol is removed through dialysis. Note: The groups except acetic acid were neutral (pH 7–7.4). E) Thermorheological response of neutral ColNB formulations at different degrees of functionalization. Here, only the low DoF groups demonstrated physical cross‐linking response at neutral pH indicated by increase in storage moduli *G*′.

We used thiol‐norbornene click chemistry for the rapid printing of constructs using two separate deep vat printing approaches: tomographic and FLight printing. Here, based on existing studies,^[^ ^35^, ^36^ ^]^ we functionalized collagen with norbornene groups (**Figure** **2**A) through a reaction with carbic anhydride (CA) under controlled temperature (4 °C) and pH 9. We varied the amount of CA relative to the weight of the Col I in the reaction mixture (6:1, 2:1, 0.5:1; mg of CA: mg of Col I) to be able to derive different degrees of norbornene functionalization in Col I. Successful functionalization was confirmed through proton nuclear magnetic resonance spectroscopy (^1^H NMR), with a resulting degree of substitution of 0.481 mmol mg^−1^ (low DoF; achieved through a CA:Col I ratio of 0.5:1), 1.323 mmol mg^−1^ (moderate DoF; achieved through a CA:Col I ratio of 2:1), and 2.25 mmol mg^−1^ (high DoF; achieved through a CA:Col I ratio of 6:1) mmol g^−1^, respectively. As for the thiolated crosslinkers, 4PEGSH was commercially procured (Sinopeg), while GelSH was synthesized based on our previous work.^[^ ^37^ ^]^ Using ^1^H NMR analysis, the resulting degree of substitution of thiol groups in GelSH was 0.774 mmol g^−1^ (Figure S1, Supporting Information). To enable comparison between ColNB and pure gelatin‐based matrices, we also norbornene‐functionalized gelatin (GelNB) at varying DoFs, following our previously established method.^[^ ^20^ ^]^ The degree of functionalization was quantified via ^1^H NMR and found to be 2% (low DoF), 14% (medium DoF) and 61% (high DoF), respectively. To determine the effect of norbornene functionalization on the secondary protein structure of both native and modified collagen, we used circular dichroism (CD) spectropolarimetry. The native collagen (positive control), and the three ColNB formulations displayed a characteristic pattern of triple helical conformations with positive bands at 220 nm (Figure 2B,C). Here, the norbornene functionalization slightly attenuated the positive peak at 220 nm and negative peak at 198 nm, which is consistent with existing studies.^[^ ^35^, ^36^ ^]^ As a comparison, the characteristic peak at ∼220 nm was absent in the spectrum of pure gelatin, confirming the loss of secondary structure upon denaturation (Figure 2B). Notably, we also recorded CD spectra of GelNB at different degrees of norbornene functionalization (see Experimental Section for details) and confirmed the absence of the peak at 220 nm owing to the absence of triple helix structures within gelatin (Figure S2, Supporting Information).

Typically, pristine Col I solutions are stored in acidic conditions (pH 3–4), and when neutralized (pH 7–7.4) prior to adding cells, the Col I formulations undergo physical cross‐linking via disordered fibrillogenesis.^[^ ^38^, ^39^ ^]^ This cross‐linking is mediated via hydrogen bonded water‐clusters between the constitutive triple helices, a process which is faster at higher temperatures.^[^ ^40^ ^]^ This makes handling of such resins time and temperature sensitive. However, it has been demonstrated that chemical modification using methacrylate or norbornene groups can shift the isoelectric point of the modified Col I, thereby affecting its physical gelation at neutral pH.^[^ ^35^ ^]^ Accordingly, we first investigated the rheological response of neutral formulations of pristine Col I and ColNB (low, medium, and high DoF) at different temperatures (4, 24, and 37 °C; henceforth termed as thermorheology tests). As expected, the pristine Col I formulations demonstrated physical cross‐linking (Figure 2D), resulting in an increase in storage modulus upon increasing temperature. However, amongst the different ColNB formulations, only the ones with a low DoF demonstrated limited physical cross‐linking, while the medium and high DoF formulations did not undergo an increase in storage modulus (Figure 2E), which is also consistent with existing studies.^[^ ^35^, ^36^ ^]^

Notably, the physical gelation properties of pristine or modified Col I (e.g., the low DoF ColNB) can also be affected by additives such as polyols which can temporarily inhibit water‐mediated hydrogen bonding.^[^ ^40^ ^]^ This can, in turn, allow easy processing of the resins, and subsequently the retrieval of the constructs. As we highlight later, our resins formulations contained iodixanol, which is a biocompatible non‐ionic radiocontrast agent used for refractive index (RI) matching.^[^ ^41^ ^]^ Interestingly, iodixanol can allow inhibition of water‐mediated hydrogen bonding at neutral pH in the pristine Col I formulations (i.e., inhibit the fibrillogenesis of Col I, Figure 2D). Furthermore, this effect is temporary, and the pristine Col I formulations regain their physical cross‐linking potential upon removal of iodixanol via dialysis (Figure 2D). Using CD measurements, we also verified that the addition of Iodixanol in Col I and ColNB does not affect the secondary protein structure of the collagen formulations, i.e., the groups exposed to iodixanol showed similar ellipticity, (Figure 2C).

**Figure 3:**
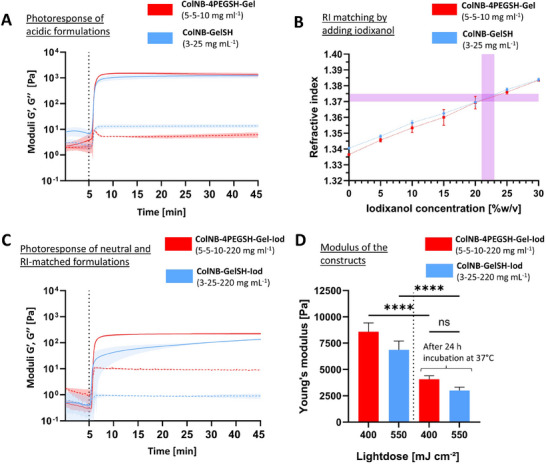
Photoresponse of the resin formulations. A) Photorheological response of acidic resin formulations with GelSH or 4PEGSH as crosslinkers (solid and dotted lines represent storage (*G*′) and loss moduli (*G*″), respectively). B) Addition of Iodixanol for the RI matching of the materials. An iodixanol concentration of 22% w/v was found to be optimal to allow matching of RI to that of cells (1.37–1.375).^[^ ^41^ ^]^ C) Photorheological response of neutralized resin formulations with GelSH or 4PEGSH as crosslinkers and with Iodixanol (Iod) as the RI matching agent containing iodixanol featuring a lower storage modulus than the constructs without iodixanol. D) Modulus of the constructs (made using the optimal light doses) immediately after fabrication and after incubation in PBS for 24 h at 37 °C. Data represented as mean ± SD (*n* = 3), statistical significance was determined by one‐way ANOVA and is denoted as follows: ^****^ represents *p* < 0.0001, ^***^ represents *p* < 0.001.

To be able to optimize the resin formulations which could be used in both tomographic and FLight printing, we performed initial screening experiments using high DoF ColNB formulations, as they enabled the fastest photo‐response when comparing the three resin formulations (Figure S3, Supporting Information). Of the two techniques, tomographic printing necessitates the use of resins exhibiting high viscosity or ideally thermo‐reversible gelation, to prevent part sedimentation during printing.^[^ ^1^, ^43^ ^]^ While the formulations containing GelSH can undergo thermo‐reversible gelation at 4 °C, the ones with 4PEGSH as the crosslinker do not undergo such a gelation. Therefore, we added 1% w/v (i.e., 10 mg ml^−1^) of sacrificial gelatin in the formulations containing 4PEGSH, which allowed printing in a thermally gelled state when cooled to 4 °C. To facilitate comparison between the two techniques, we used the same material constitutions for both tomographic and FLight printing. First, we optimized the concentrations of ColNB, 4PEGSH, and GelSH to achieve similar storage moduli between the two types of resins (i.e., those using GelSH or 4PEGSH as crosslinkers) upon photo‐crosslinking. **Figure** **3**A shows the selected formulations (pH 3–4) which demonstrated a photoclick response and resulted in similar storage modulus (≈1 kPa) after photo‐crosslinking. Subsequently, neutralized formulations (pH 7.2–7.4) were developed using the optimized material constitutions (from Figure 3A) to allow the addition of cells. We added iodixanol in each resin formulation to increase the RI, which reduces light scattering due to the encapsulated cells, thereby allowing high resolution prints.^[^ ^41^ ^]^ Accordingly, an iodixanol concentration of 220 mg ml^−1^ (22% w/v) was found to be optimal (Figure 3B) to increase the RI of each type of resin to match the used cells (RI = 1.37–1.375).^[^ ^41^ ^]^ The photorheological response of the neutralized and RI‐matched resins demonstrated lower storage moduli (≈200 Pa). This is consistent with existing studies which have shown that the presence of iodixanol can reduce the cross‐linking efficiency and hence the storage modulus of the constructs.^[^ ^41^, ^44^ ^]^ Based on the optimized neutral and RI‐matched formulations, we investigated the photorheological response of the resin high DoF formulations based on GelSH or 4PEGSH as crosslinkers (Figure 3C), where both resin formulations demonstrated a rapid photoclick response. Figure 3D shows the results of mechanical testing for ColNB formulations with 4PEGSH or GelSH as crosslinker. Here, the constructs were fabricated (FLight printed) using the optimized light dose conditions (light doses were optimized via dose tests (Figure S4, Supporting Information) based on our previous work.^[^ ^11^, ^44^ ^]^. Immediately after fabrication, the average stiffness of the constructs was 7.8 ± 0.68 and 6.3 ± 0.74 kPa (not statistically significantly different) for the constructs based on the 4PEGSH and GelSH as crosslinker, respectively. After incubation for 24 h at 37 °C, the stiffness was reduced to ≈3.6 ± 0.43 and ≈2.8 ± 0.23 kPa for the two formulations (not significantly different from each other, but significantly different to as printed), which can be attributed to the release of the 22% w/v iodixanol from both the formulations and the release of the sacrificial gelatin in constructs with 4PEGSH as cross‐linker (release kinetics shown in Figure S5, Supporting Information).

**Figure 4:**
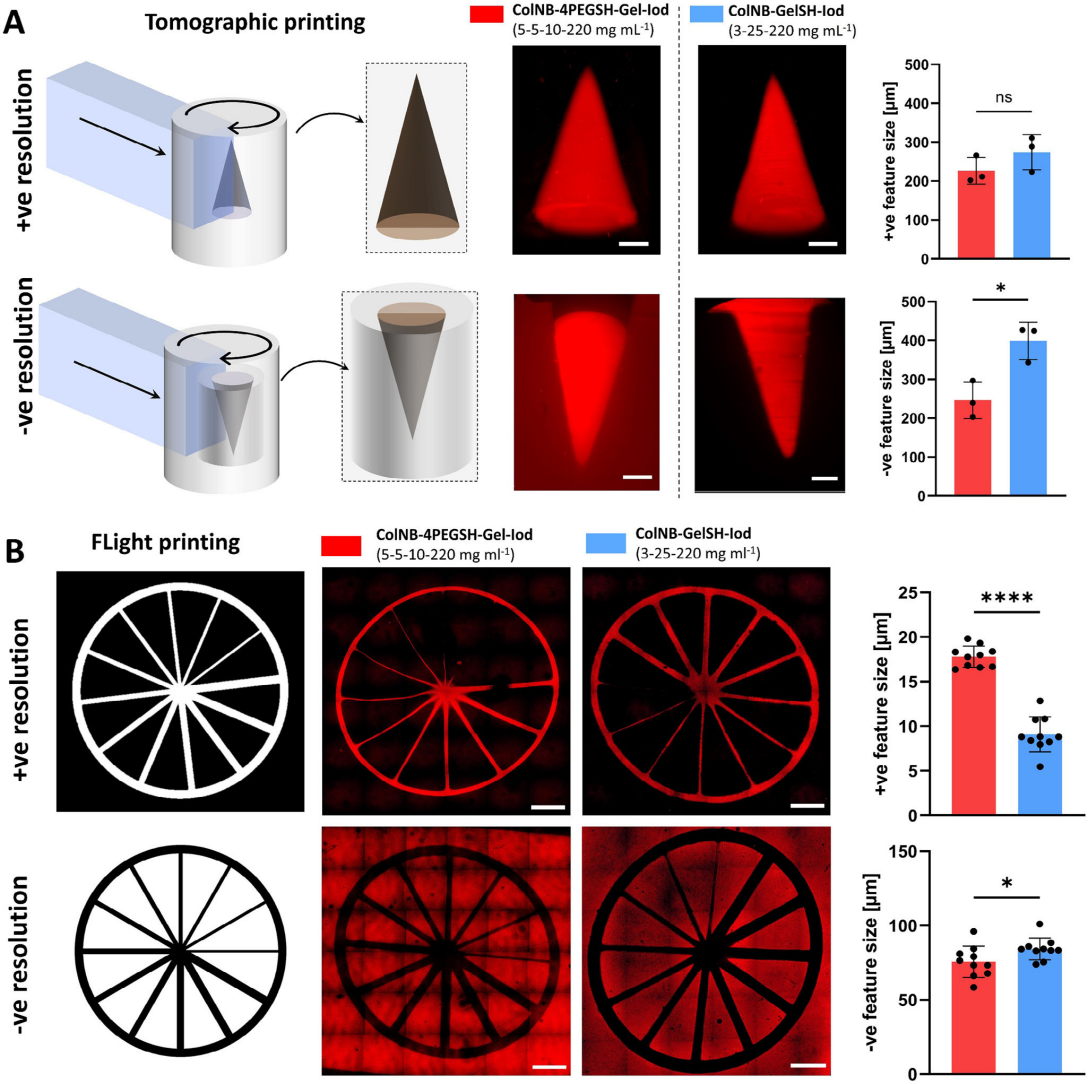
Characterization of print resolution. A) Tomographically printed constructs for positive (+ve) and negative (−ve) resolution (dotted lines represent region of capture during light sheet microscopy). The outcomes of the resolution tests (i.e., the minimum feature size) are shown on the right. Note: in the negative resolution, fluorescently labeled resin was perfused in the hollow cone to visualize the features. All scale bars are 1000 µm. B) Spoke wheel patterns to characterize the resolution via FLight printing (top‐down projection in µ‐slide containing the photoresins). The graph on the right shows the outcome of the resolution test. Data represented as mean ± SD (VP: 3 individual constructs, FLight: 10 measurements), statistical significance was determined by unpaired *t*‐test and is denoted as follows: ^****^ represents *p* < 0.0001, ^*^ represents *p* < 0.05.

Tomographic and FLight printing processes have different process capabilities in terms of complexity of the prints and achievable resolution.^[^ ^19^ ^]^ Here, the tomographically printed constructs with 4PEGSH as the crosslinker demonstrated a positive and negative printing resolution of ≈220 µm, while the constructs with GelSH as the crosslinker featured ≈275 µm and 400 µm as positive and negative resolution, respectively (**Figure** **4**A). For the FLight printed constructs (Figure 4B), the resolution (both positive and negative resolution) of constructs based on GelSH or 4PEGSH as crosslinkers were comparable to each other, but the positive resolution (10–20 µm) was generally better compared to the negative resolution (50–100 µm). Between the two approaches, FLight printing enabled higher resolution, which is likely due the light projection coming from a single direction, which can reduce non‐specific cross‐linking in the resin formulations. In contrast, to achieve the cumulative light dose in a tomographic setting, the rotation of the vial emulates a multi‐direction light projection, possibly causing non‐specific cross‐linking. Another contributing factor can be the optical self‐focusing effect in FLight printing, which leads to the light being guided into the already‐crosslinked material in the form of microfilaments, thereby reducing scattering and improving resolution.

**Figure 5:**
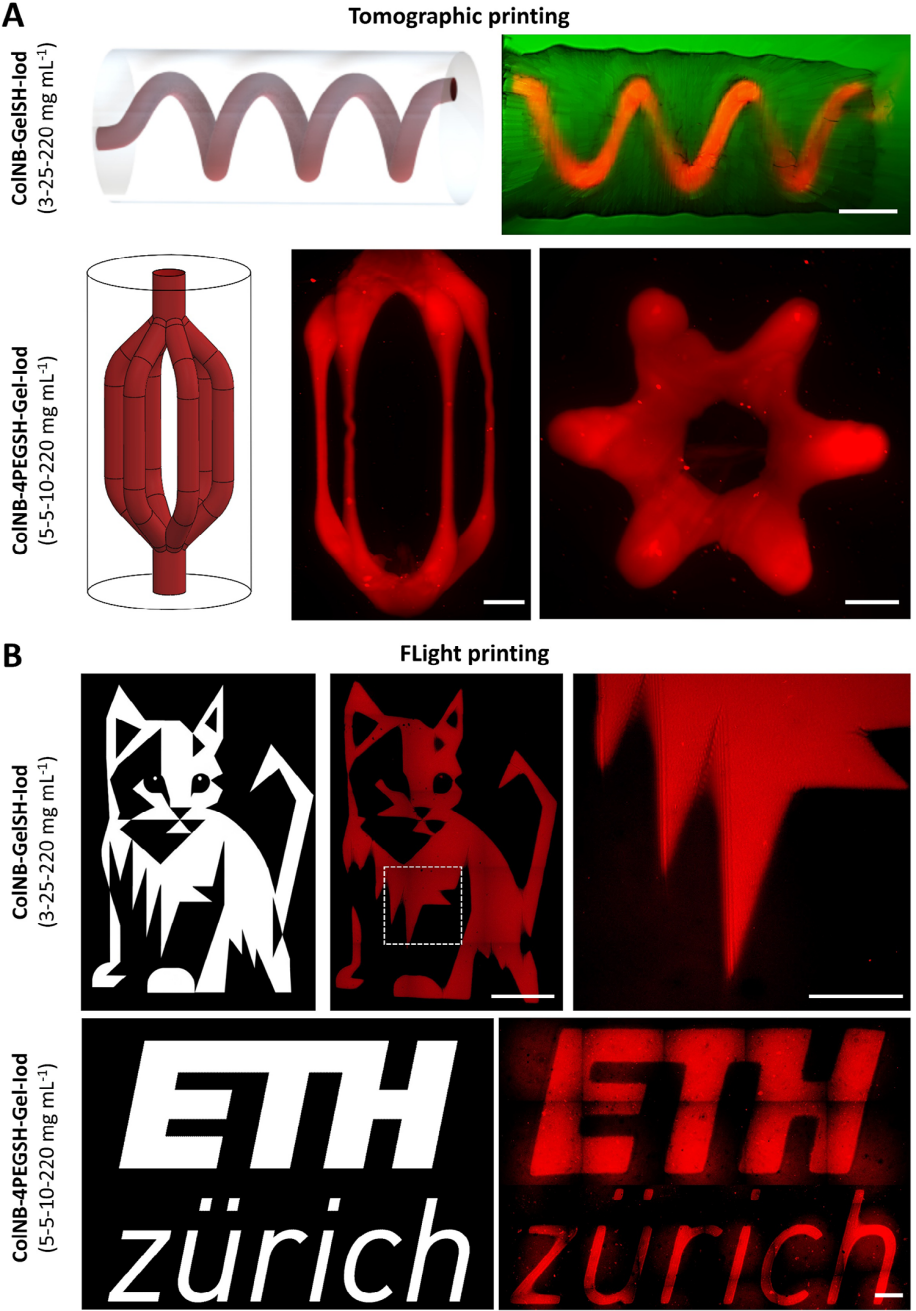
Complex constructs printed with tomographic or FLight printing. A) The top two panels show tomographically printed perfusable constructs with GelSH (spiral channels) or 4PEGSH (bifurcating channels) as the crosslinkers. The perfused media comprised of rhodamine‐labeled gelatin methacrylate (GelMA), which was crosslinked within the constructs after perfusion. B) Bottom two panels show stitched confocal images of FLight constructs with GelSH (cat model) or 4PEGSH (ETH logo) as the crosslinkers. Scale bars of tomographically‐ and FLight‐printed constructs are 1000 and 100 µm, respectively.

Despite higher resolution, one must note that FLight printing, in its current stage of development, is still a 2.5D approach (i.e., the constructs feature the same cross‐section throughout their entire thickness).^[^ ^1^ ^]^ In contrast, tomographic printing allows the fabrication of complex 3D shapes, such as the perfusable constructs shown in **Figure** **5**A. These constructs were made with GelSH or 4PEGSH as crosslinkers, and have relevance toward 3D bioprinting, especially for the rapid fabrication of vascularized tissue models.^[^ ^45^ ^]^ Notably, the thin channels in the center of the bifurcating channel‐structures (Figure 5A; formulations with 4PEGSH as the crosslinker) can be attributed to inhomogeneity in the deposited light dose typically associated with tomographic printing.^[^ ^46^ ^]^ In contrast, FLight printing can enable 2.5D prints with complex cross‐sections (example prints of a cat image or ETH logo with GelSH or 4PEGSH as crosslinkers, respectively, are shown in Figure 5B).

**Figure 6:**
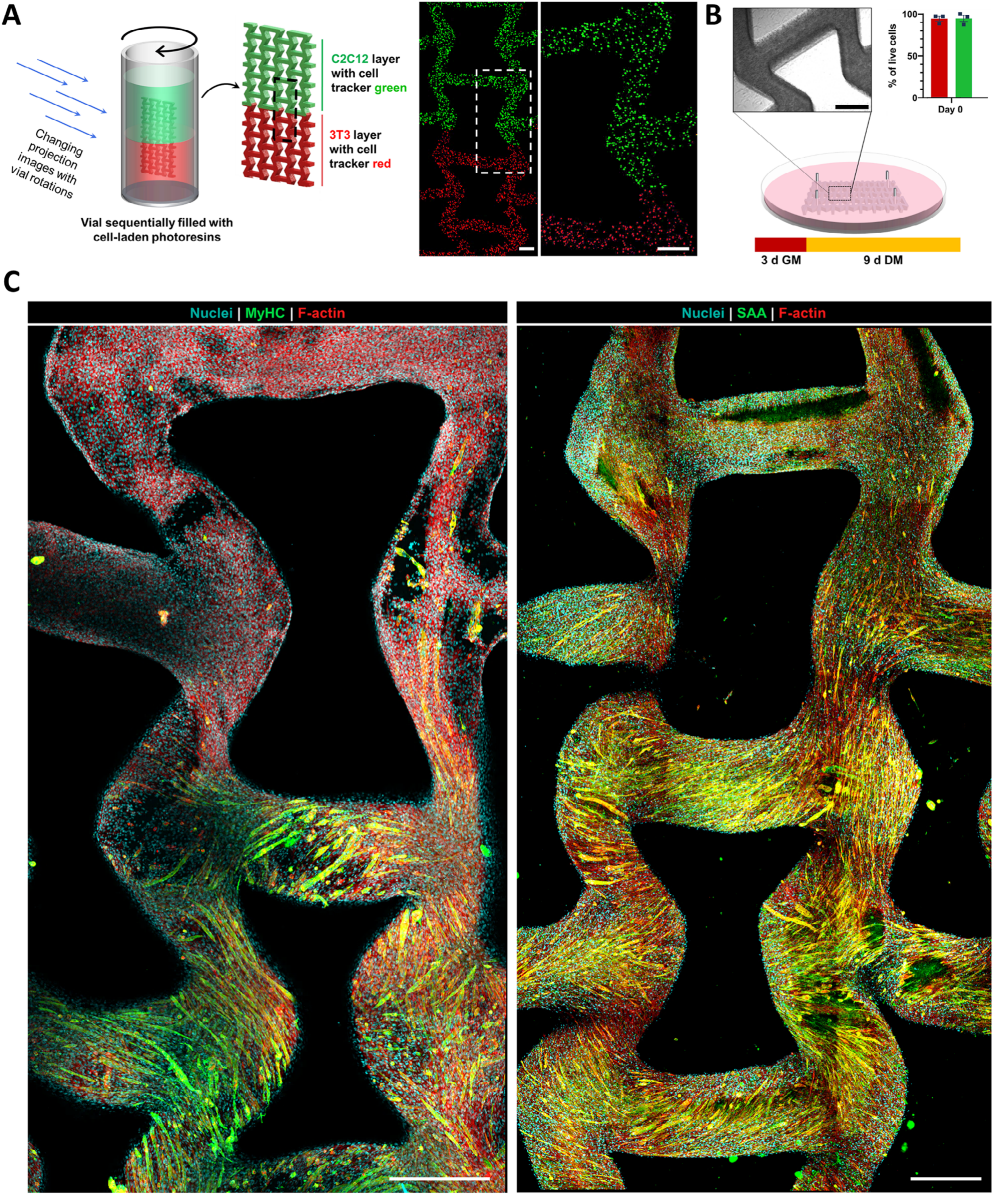
Tomographically printed muscle‐connective tissue constructs and their characteristics upon maturation. A) Tomographic printing was used to create bi‐material constructs featuring 3T3 fibroblasts (labeled with cell tracker red) and C2C12 cells (labeled with cell tracker green) in the top and bottom layers, respectively. Scale bars are 250 µm. B) The constructs featured a cell viability > 90% (green and red bars represent 3T3 and C2C12 cells, respectively), and were pinned at the vertices to prevent warping and were cultured for 3 days in growth media and 9 days in differentiated media. C) Image maps of the constructs after maturation, where both regions demonstrated cell proliferation throughout the constructs, and the C2C12 regions demonstrated myosin heavy chain (MyHC) staining and sarcomeric alpha actinin (SAA) staining, which are characteristics of maturated muscle constructs. Scale bars of the image maps are 500 µm.

As tomographic or FLight printing methods present their unique advantages and limitations, it is important to consider the specific application that these approaches are being used in. Accordingly, in subsequent studies, we investigated multi‐material tissue printing, specifically, the fabrication of a muscle‐connective tissue interface model, and compared the two printing approaches in terms of the complexity of the printed constructs, and the biomimicry one could achieve with the two approaches. The procedure for the fabrication of the multicellular tissue constructs (a muscle‐connective tissue interface model) via tomographic printing is illustrated in **Figure** **6**A. In this approach, we used C2C12 myoblasts for the muscle region, and 3T3 fibroblasts for the connective tissue region. To facilitate visualization of the transition zones in the interface model, the C2C12 and 3T3 were labeled with cell tracker green and red, respectively. In all cellular studies, only the GelSH‐based resins were used, and the cells were added at a concentration of 10^6^ cells mL^−1^. To prepare the printing vial, the resins were added sequentially, with 4 °C incubation steps in‐between to enable a clear transition between the two layers. Since the base material for the two layers was the same, a single light dose was employed for the fabrication of the (10 × 8 × 1.5 mm) constructs (Figure 6A). We used auxetic meshes based on our previous work,^[^ ^19^, ^47^ ^]^ to show the freedom of geometry which is enabled by the tomographic printing approach. After fabrication, the constructs were pinned at the vertices (Figure 6B) to prevent warping during the culture time of 12 days (3 days growth + 9 days differentiation) followed by fixation and immunostaining. Post printing, the cell viability for both, 3T3 and C2C12 was above 90% (Figure 6B).

Figure 6C shows the mapped image of the immunohistochemically stained samples after maturation. Here, we labeled the nuclei, f‐actin, and either anti‐myosin heavy chain (MyHC) or sarcomeric alpha actinin (SAA). Within the maturated constructs, the expression of MyHC and SAA is highly localized in the regions with C2C12 cells (i.e., the bottom half of the constructs). Further, the orientation of the myotubes (as seen from the MyHC staining) is not uniaxial and is more likely along the mechanical loading introduced by pinning the constructs. This is likely due to the absence of well‐defined, uniaxial cell‐guiding microarchitectures within the constructs. This is an aspect which has been addressed in the subsequent study, where we investigated FLight printing for the fabrication of these interface models.

An important advantage of FLight printing is the emergence of microfilaments, which act as excellent cell guidance cues for the alignment and proliferation of cells, making this approach highly suitable for the fabrication of anisotropic tissues. Prior to creating the muscle‐connective tissue interface model using FLight printing, we were interested in comparing the suitability of the photoclickable ColNB‐based resin formulations in our present work with GelNB‐based formulations we have demonstrated previously for muscle biofabrication.^[^ ^11^ ^]^ To ensure optimal material comparison, both matrices were prepared with equivalent protein concentrations, i.e., the ColNB‐GelSH resins at 0.3–2.5% w/v were compared to GelNB‐GelSH resins at 1.4–1.4%w/v. For these experiments, medium DoF GelNB was used.^[^ ^20^ ^]^ Furthermore, we added iodixanol within the GelNB‐GelSH formulations to match the refractive index to that of the ColNB‐GelSH resin. To visualize the microstructure both matrices were fluorescently labeled with rhodamine (Figure S6A, Supporting Information). Both materials were photocrosslinked to a comparable Young's modulus of ≈6 kPa, which required a dose of 350 mJ cm^−^^2^ for GelNB‐GelSH and 525 mJ cm^−^^2^ for ColNB‐GelSH hydrogels. We further analyzed the microarchitecture and found filament diameters ranging from 1.8 to 15 µm in both resin systems. Notably, ColNB‐GelSH formulations exhibited a broader distribution of filament and microchannel diameters, suggesting a more heterogeneous internal structure (Figure S6B, Supporting Information). Within the cross‐linked constructs, we hypothesized that collagen‐based matrices would enhance cell–matrix interactions by facilitating engagement with a broader array of integrins, specifically collagen‐binding integrins (α1β1, α2β1, α10β1, and α11β1), which recognize the GFOGER sequence of collagen's triple helix.^[^ ^31^ ^]^ Accordingly, we encapsulated C2C12 myoblasts in the two hydrogel systems and stained for integrin β1 (ITGB1) expression normalized to the cell count within the hydrogels. Analysis of immunofluorescence staining confirmed that that ITGB1 expression increased over culture time in both hydrogels, with cells encapsulated in ColNB‐based gels consistently exhibiting higher overall ITGB1 expression at all measured time points (Figure S7, Supporting Information). In contrast, the ITGB1 expression in GelNB‐based gels decreased during the first five days of culture, potentially due to the lack of optimal biological or chemical cues compared to collagen.^[^ ^31^ ^]^ After a week, the ITGB1 expression increased, presumably resulting from matrix deposition and remodeling by the C2C12 cells.

**Figure 7:**
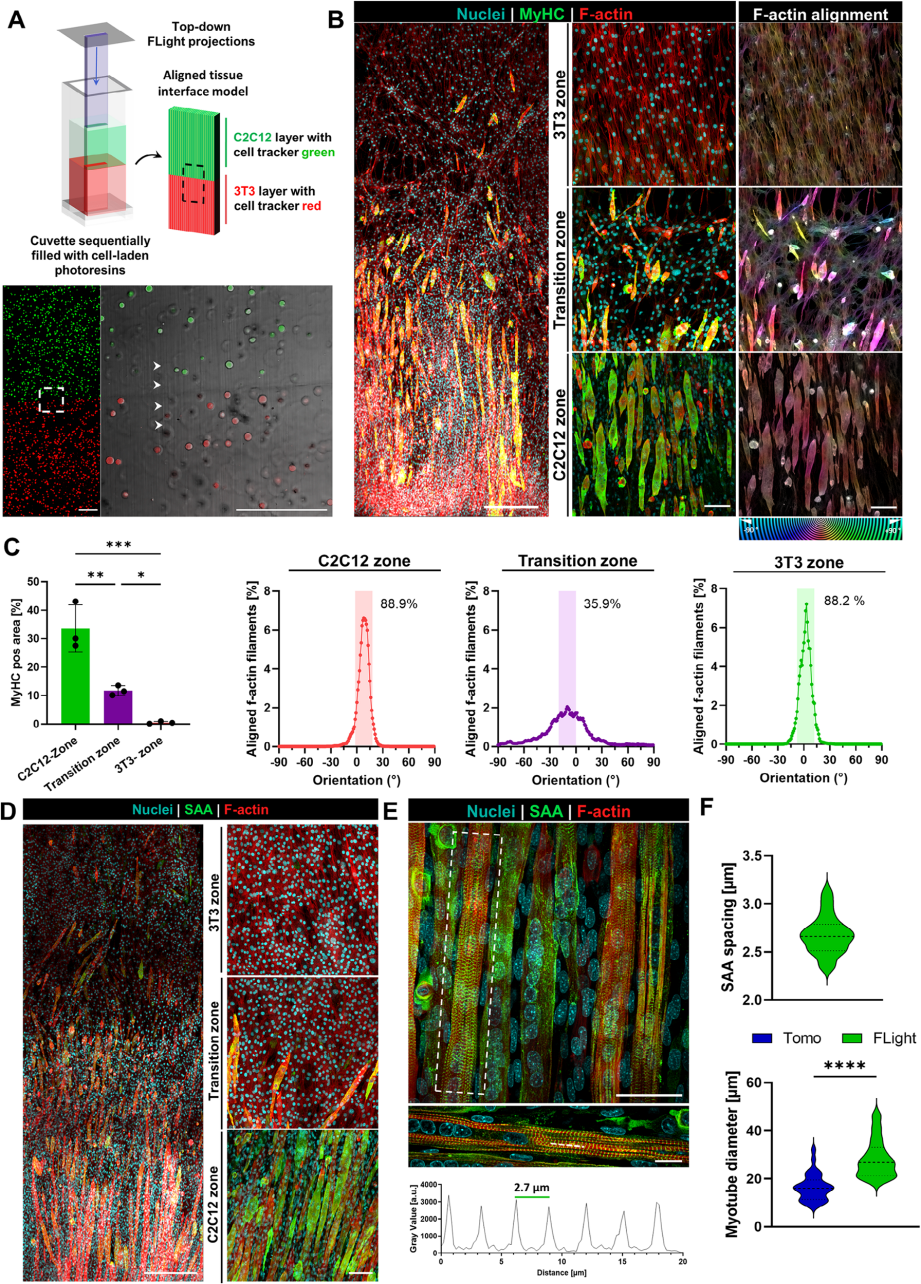
Flight printed constructs featuring the muscle‐connective tissue interface and their maturation characteristics. A) FLight printing was used to create bi‐material constructs featuring 3T3 and C2C12 cells labeled with cell tracker red and green, respectively. The white arrows in the micrographs represent the microfilaments generated through FLight printing. Scale bars are 250 µm. B) Constructs after maturation (3 days growth + 9 days differentiation) demonstrated an aligned cellular morphology, with myotubes expression myosin heavy chain (MyHC staining) gradually through the constructs (i.e., increasing through the transition and muscle zones), enabling improved biomimicry compared to the VP constructs. A colormap of the f‐actin alignment is shown on the right for each zone (C2C12, Transition or 3T3 zones). Scale bars of the maps are 250 µm, while the scale bars in crop‐in images are 100 µm. C) Quantification of Myosin heavy chain signal and analysis of f‐actin alignment across different regions. Colored boxes indicate the summed proportion of aligned actin filaments within ±10° of the dominant orientation peak. Data represented as mean ± SD (*n* = 3), statistical significance was determined by one‐way ANOVA and is denoted as follows: ^***^ represents *p* < 0.001, ^**^ represents *p* < 0.01, and ^*^ represents *p* < 0.05. D) Maturated constructs demonstrate an aligned cellular morphology, with myotubes expression sarcomeric alpha actinin (SAA staining) gradually through the constructs (i.e., increasing through the transition and muscle zones. Scale bars of the maps are 250 µm, while the scale bars in crop‐in images are 100 µm. E) Crop‐in of C2C12 laden region demonstrating highly organized sarcomere structures, and corresponding analysis of selected region (inset image on bottom) demonstrates a regular sarcomere spacing of 2.68 µm. Scale bars: 50 µm, while the scale bars in crop‐in images are 20 µm. F) The FLight constructs also demonstrate higher myotube diameter compared to the tomographically printed constructs. Data represented as mean ± SD (*n* = 30) and statistical significance is denoted as follows: ^****^ represent *p* < 0.0001.

As previously highlighted, the filaments in FLight printing approach are generated along the direction of propagation of the light beam, as such limiting the freedom of geometry in the lateral direction (i.e., direction perpendicular to the projection direction). Nevertheless, we used a top‐down FLight projection into photoresin‐laden cuvettes (illustrated in **Figure** **7**A), to fabricate muscle‐connective tissue interface models featuring C2C12 and 3T3 cell layers labeled with cell tracker green and red, respectively. Here, we used the same resin formulations and preparation steps as those in the tomographic printing results (Figure 6A), however, instead of projecting an auxetic mesh design, we projected a simple rectangular image (4 × 1 mm) into the resin‐filled cuvettes. This allowed the fabrication of rectangular constructs (10 × 4 × 1 mm) with the two distinctly divided cell layers (Figure 7A). Notably, the cell‐guiding microfilaments were prevalent throughout the two layers, which promoted cell alignment, migration and proliferation between the two layers during the culture phase.

After maturation, the tissue constructs demonstrated aligned cell layers in both the 3T3 and C2C12 compartments, as well as alignment of MyHC and f‐actin with multinucleated myotubes in the muscle region (Figure 7B). Immunofluorescence staining revealed a significant, gradual increase of MyHC expression from the 3T3 region – characterized by a negligible MyHC positive area – to the C2C12 region where up to 40% of the area was MyHC positive in the observed confocal sections (Figure 7C). Notably, the alignment of cellular f‐actin was high in the peripheral regions, with ≈90% of actin filaments oriented within ±10° of the predominant orientation angle. In contrast, the transition zone exhibited a markedly broader distribution of f‐actin orientations, indicating reduced anisotropy and cellular alignment, which has also been observed in studies on biofabrication of myotendinous junctions.^[^ ^48^, ^49^ ^]^ Importantly, despite clear demarcation in the C2C12‐ and 3T3‐laden regions within the biofabricated constructs (Figure 7A), the MyHC after tissue maturation was also present in the transition zones between the two cell regions (Figure 7B,C). This is likely due to the presence of microchannels between the microfilaments, which have been shown to actively promote cell guidance, migration and proliferation.^[^ ^11^ ^]^ This is highly relevant toward tissue interface printing, as most tissue interfaces, such as a muscle‐tendon interface, feature an interspersed distribution of the fibers and cells constituting the two tissue types.^[^ ^50^, ^51^ ^]^

Within the FLight constructs, in addition to the gradual transition of muscle‐specific extracellular matrix organization, we also observed contractility in the regions featuring C2C12 cells, and no contractility in the regions with the 3T3 cells (Video S1, Supporting Information). This can be attributed to the emergence of organized sarcomere structures within myotubes which allows spontaneous contraction of the muscle tissue (Figure 7D,E). The average sarcomere length (i.e., spacing between adjacent z‐bands in the sarcomere structure) of 2.68 ± 0.2 µm also mimics what is typically found in mouse muscle (Figure 7F).^[^ ^52^, ^53^ ^]^ Moreover, the myotube diameter was also higher in the FLight constructs (28.4 ±7.8 µm) compared to the tomographically printed constructs (16.5 ± 5.5 µm, Figure 7F). The Flight constructs closely resemble the myotube diameter found in existing studies based on casting,^[^ ^54^, ^55^, ^56^ ^]^ or micropatterning,^[^ ^57^, ^58^ ^]^ as well as the native mouse muscle,^[^ ^59^ ^]^ indicating the suitability of this technique in producing biomimetic muscle constructs. In comparison, organized sarcomere structures and contractile behavior were absent in the tomographically printed constructs shown in Figure 6C at the same timepoints. This is likely because the tomographically printed constructs lack the cell‐guiding microfilaments along a uniaxial orientation, which would otherwise favor cell proliferation within microchannels and facilitate the formation of thicker myotubes and a well‐defined sarcomere structure within such constructs.

## 3 Discussion

We have demonstrated collagen‐based photoclickable resins which can be effectively used in deep vat bioprinting techniques for the fabrication of complex multicellular tissues. While gelatin‐based resins such as methacrylated gelatin (GelMA) or norbornene and thiol‐functionalized gelatin have largely dominated the field of tissue fabrication using DVP,^[^ ^5^, ^11^, ^20^, ^41^ ^]^ resins based on collagen offer unique advantages in terms of enhanced bioactivity and biomimicry of the physiological environment of the extracellular matrix.^[^ ^39^, ^60^ ^]^ In this work, we utilized norbornene‐functionalized collagen and combined it with 4PEGSH and GelSH as crosslinkers. In the future, to be able to claim a purely photoclickable collagen resin formulation, ColNB in conjunction with thiolated collagen (ColSH) will be explored.^[^ ^61^, ^62^ ^]^ Notably, methacrylation of collagen,^[^ ^63^, ^64^ ^]^ albeit chain growth polymerization, also offers a single material system for the photopolymerization, which has not yet been explored with deep vat printing. However, it is important to control the degree of methacrylation, as excessive methacrylation may affect the triple helical conformations in collagen, as observed previously in CD measurements.^[^ ^35^ ^]^ In the present work, norbornene functionalization still maintained the triple helix conformation in the Collagen resins, thus retaining the bioactivity of the resin formulations (Figure 1C). Given that norbornene‐functionalization is a widely applicable method requiring only free amine groups, it presents exciting opportunities for future research, where the functionalization approach could be extended to other members of the collagen family.^[^ ^65^ ^]^ For instance, in the context of cartilage tissue engineering, norbornene‐functionalized collagen type II could be explored.^[^ ^66^ ^]^ Furthermore, this approach opens the possibility for creating tissue‐specific, light‐sensitive biomaterials by functionalizing dECM with norbornene groups compatible for DVP.^[^ ^32^, ^67^ ^]^ The dECM hydrogels exhibit, in addition to a blend of different types of collagen (Col I, Col II, etc.),^[^ ^68^, ^69^ ^]^ an environment rich in glycosaminoglycans and growth factors to boost cell survival and differentiation.^[^ ^33^, ^70^ ^]^ As a proof‐of‐concept demonstration, we have performed additional tests on norbornene functionalization of dECM (i.e., dECM‐NB) formulations which were derived from the porcine skeletal muscle based on the work by the Christman group (see Experimental Section for details).^[^ ^70^, ^71^, ^72^ ^]^ The procedure for the functionalization was the same as that of the ColNB formulations. Selected photoresin formulations comprising of dECM‐NB and GelSH demonstrated rapid photoclick response, high fidelity printability (using FLight projection), as well as high cell viability after printing (see Figure S8, Supporting Information). Future studies could characterize the matrix composition of the modified dECM and compare these dECM‐based resins to those based on collagen I or other collagen blends for obtaining the most suitable resin formulations for musculoskeletal tissue engineering.

We demonstrated that the addition of iodixanol not only allows for RI matching, but importantly, can also temporarily inhibit the physical cross‐linking of pristine collagen or low DoF ColNB. This could be useful for future applications utilizing modification‐free cross‐linking of collagen formulations (similar strategies as the dECM formulations).^[^ ^32^, ^73^ ^]^ Printing with pristine collagen also improves the translational potential of the biomaterial grafts.^[^ ^26^, ^74^ ^]^ Notably, for acellular prints, the collagen formulations can also be printed in acidic conditions, obviating the need for the addition of iodixanol in any formulation. We have verified the same by tomographically printing the ColNB resins with both 4PEGSH and GelSH crosslinkers under acidic conditions (see Figure S9, Supporting Information).^[^ ^50^, ^51^ ^]^ Also, while FLight printing was more suitable for the printing of the muscle‐connective tissue interface models, it is to be noted that tomographic printing may be highly suitable for the printing of 3D complex tissues such as vascularized models (Figure *5*A).^[^ ^75^, ^76^ ^]^ Even for tomographically printed tissues, stimulation of the maturing tissues via electrical and mechanical means can be used to guide the polarization and alignment of cells and the cell‐secreted matrix.^[^ ^77^, ^78^ ^]^ We observed in the tomographically printed constructs that cells aligned along mechanical loads exerted by contracting myoblasts on the pinned constructs. Notably, the C2C12 myoblast‐laden re‐entrant honeycomb structures reduced in size during tissue culture due to their contractile nature (Figure 6C). Another important consideration is the duration of culture. In this work, tissues were cultured only for 12 days; however, prolonged tissue culture may lead to improved muscle biomimicry (i.e., synchronous contractility and increased muscle fiber diameter) in the muscle regions.^[^ ^79^ ^]^

While the obvious use of engineered tissues is toward the repair or replacement of damaged tissues, their demand has also been dramatically fueled by recent efforts to reduce, refine, and replace (3R) the use of animals in research.^[^ ^80^, ^81^ ^]^ Recent studies have demonstrated that up to 50% of pre‐clinical animal studies are non‐reproducible, and more than 90% of the drugs, which were demonstrated to be pre‐clinically safe and effective in animal trials, eventually fail in human clinical trials.^[^ ^82^ ^]^ Researchers and clinicians are therefore questioning the relevance of such pre‐clinical trials, and earlier this year, the Food and Drug Administration (FDA) of USA has lifted the requirement for animal studies before human clinical trials, if the drug can be proven safe and effective within in vitro tissue models.^[^ ^83^ ^]^ Both tomographic and FLight printing, in conjunction with photoclick chemistry, have proven to be effective approaches for the rapid and scalable fabrication of engineered tissue models. These models can serve as excellent tools to study tissue interfaces and investigate the effects of therapeutics, potentially reducing or completely removing animal usage in preclinical testing.^[^ ^50^, ^84^ ^]^

## 4 Conclusion

The use of collagen‐based resins in state‐of‐the‐art biofabrication techniques such as Deep Vat printing represents a promising advancement for the field of tissue engineering and regenerative medicine. In this work, we demonstrated collagen‐based resins based on thiol‐ene click chemistry used in conjunction with tomographic and FLight printing for the rapid and facile biofabrication of multicellular tissue models. In these resins, the addition of iodixanol enabled matching of the refractive index with cells, and also improved the resin stability by temporarily inhibiting the fibrillogenic potential of collagen at neutral pH. While we had used 4PEGSH and GelSH as crosslinkers in the present work, future work could focus on the use of thiolated collagen (for resin based purely on collagen) as crosslinkers, or the use of the native tyrosine groups for cross‐linking (for printing using pristine unmodified collagen). In terms of printing muscle‐connective tissue interface models, while tomographic printing allowed more complex architectures to be printed (e.g., auxetic meshes), FLight printing allowed enhanced biomimicry through the formation of aligned multinucleated myotubes with sarcomere structures resembling native muscle tissues. In addition to prolonging the duration of culture, for achieving biomimetic muscle fiber diameter and synchronous contractility, future work will investigate the hybridization of the two techniques for increased tissue complexity.

## 5 Experimental Section

### Matrix Synthesis

Collagen‐Norbornene (ColNB) Synthesis. Bovine Type I Collagen (Pure Col, Advanced Biomatrix) was dissolved at 4 °C in 20 mmol acetic acid at 1 mg mL^−1^, after complete solution the PH was set to 9 by the addition of 1 m NaOH (sodium hydroxide). cis‐5‐norbornene‐endo‐2,3‐dicarboxylic anhydride (Carbic anhydride, CA, 6:1 w/v ratio in mg per mL solution) was dissolved in acetone (1:20 ratio in mL acetone per mg of collagen) and added dropwise. The pH was maintained at 9 for a total of 4h, and the reaction was stopped by adjusting the pH to 7.4 by the addition of 1 m HCL (hydrochloric acid). The solution was dialyzed (at 4 °C) against Milli‐Q water with frequent water changes for 5 days. The dialyzed material was lyophilized and stored at −20 °C until further use. Degree of functionalization (DoF) was determined by ^1^H‐NMR (Bruker Ultrashield, 400 MHz, 64 scans). ColNB was dissolved in acetified D_2_O+DCl solution (Apollo Scientific) containing 0.5 mg mL^−1^ 3‐(trimethylsilyl)‐1‐propanesulfonic acid (DSS), which was adopted as internal standard. In short, DSS nine methyl protons (0.2 to −0.2 ppm) were compared to the norbornene protons (6.325 to 6.225 ppm) and molarity of ‐NB groups per mg of ColNB was calculated. Importantly, by changing the carbic anhydride to collagen ratio, different degrees of functionalization could be achieved (Figure 2).

Gelatin‐thiol (GelSH) synthesis. The thiol‐modified gelatin was synthesized following our previously published method.^[^ ^37^ ^]^ Briefly, porcine‐derived (Type A) gelatin was dissolved in 0.15 m MES (2‐(N‐morpholino)ethanesulfonic acid) buffer (pH 4) at 50 °C to get a 2% w/v solution. When completely dissolved, DTPHY (3,3'‐ Dithiobis(propionohydrazide)) was added under constant stirring to reach a final concentration of 10 mmol. When completely dissolved, EDC (1‐Ethyl‐3‐(3‐dimethylaminopropyl)carbodiimide) was added to the solution (20 mmol). The reaction was then allowed to proceed at 50 °C under constant stirring for 12 h. Next, TCEP was added (30 mmol), followed by continuing the reduction reaction for 6 h. Finally, 1g of NaCl was added, and the solution was dialyzed against Milli‐Q water balanced to pH 4.5 with diluted HCl. The dialyzed material was lyophilized and stored at −20 °C until further use. DoF was again quantified by ^1^H‐NMR. Briefly, GelSH was dissolved in D_2_O containing DSS and the peaks of the standard were compared to the peaks of the DTPHY protons (2.85 to 2.75 and 2.7 to 2.6 ppm) to calculate the DoF.

Gelatin‐Rhodamine (Gel‐Rho) Synthesis. Gelatin was dissolved in 0.1 m CB (carbonate–bicarbonate) buffer (≈pH 9.0) at 37 °C to get a 1% w/v solution. Next, NHS‐Rhodamine (5/6‐carboxy‐tetramethyl‐rhodamine succinimidyl ester, 3‐fold molar excess relative to the lysine residues of gelatin) was dissolved in 1 mL of DMSO (dimethyl sulfoxide) and added to the gelatin solution to reach a final concentration of 5 mmol. The reaction was left to proceed at 37 °C overnight. The product was dialyzed against Milli‐Q water at 40 ° with frequent water changes for 5 days, lyophilized, and stored at −20 °C until further use.

Gelatin norbornene (GelNB) Synthesis: GelNB was synthesized as previously described. Briefly, gelatin was dissolved in 0.5 m carbonate–bicarbonate buffer (pH 9.0) at 50  °C to prepare a 10% (w/v) solution. Carbic anhydride (CA) was added in varying weight ratios (1:50, 1:20, 1:10) to achieve different degrees of functionalization, with intermittent pH adjustments to maintain pH 9. After five sequential additions, the pH was neutralized, and the solution was dialyzed against deionized water for 5 days with frequent water changes. The purified product was lyophilized and stored at −20 °C until use.

Preparation of decellularized extracellular matrix (dECM): The dECM was prepared following protocols established by the Christman group.^[^ ^70^, ^71^, ^72^ ^]^ Briefly, porcine loin skeletal muscle (Collagen Solutions) was trimmed of fat and connective tissue, minced, and stirred in 1% (w/v) sodium dodecyl sulfate (SDS) with antibiotics in PBS for 48 h, with frequent solution changes. During this process, the tissue turned white, indicating successful decellularization. Residual SDS was removed by extensive washing in Milli‐Q water for an additional 24 h. To remove residual lipids, the tissue was defatted using isopropyl alcohol (IPA), followed by further washing in Milli‐Q water to remove IPA traces and lyophilization. Following lyophilization, material was milled and passed through a 60 mesh screen to ensure consistent digestion. To render the material into a liquid, the dried dECM was digested in 0.1 m HCl containing 0.1% (w/v) pepsin for 48 h. The digested ECM was adjusted to pH 7.4, and then centrifuged at 10,000 rcf to remove larger particulates. The supernatant was subsequently dialyzed to remove salts, lyophilized, resuspended in PBS, sterile filtered, and finally aliquoted, lyophilized, and stored at frozen until use. Successful decellularization was confirmed by quantifying residual double‐stranded DNA, which was found to be below 5 ng mg^−1^ dry weight, and successful digestion was confirmed by performing a gelation test: a 6 mg mL^−1^ of the pre‐centrifuged dECM liquid formed a hydrogel after incubation at 37  °C for 1 h. Successful removal of SDS was confirmed using a methylene blue assay, with material having less than 10 µg residual SDS per mg of dECM. SDS polyacrylamide gel electrophoresis was used to characterize the peptide distribution in dECM, with bands being consistent with previously reported material.^[^ ^70^ ^]^ Norbornene functionalization was performed analogously to the ColNB synthesis protocol.

### Photoresin Preparation

Throughout the experiments, the final polymer concentration of the resins was maintained constant unless otherwise stated. The resins consisted of either 0.3% w/v ColNB and 2.5% GelSH or 0.5% w/v ColNB, 0.5% w/v 4PEGSH, and 1% w/v gelatin, both resins were supplemented with 22% w/v iodixanol. To prepare the bioresins ColNB was dissolved in iodixanol supplemented PBS at 0.6% w/v or 1% w/v, respectively. Similarly, the thiolated crosslinkers were dissolved in the iodixanol containing PBS at 5% w/v for GelSH and 1% w/v for 4PEGSH. Before printing, these resin components were mixed 1:1, and LAP (lithium phenyl‐2,4,6‐trimethylbenzoylphosphinate) was added to a final concentration of 0.05% w/v. Fluorescently labeled materials were formulated by adding acryloxyethyl thiocarbamoyl rhodamine B (Polysciences) to the described resins. Hollow structures were perfused with the fluorescently labeled ColNB+GelSH resin.

### Tomographic Printing

0.9 mL of the ColNB formulations (as described above) were loaded into 10 mm printing vials and left at 4 °C in the dark for 15 min to induce thermal gelation. To establish the required light‐doses needed for tomographic printing, light‐dose‐tests were conducted. Tomographic projections were performed on a commercially available printer (Tomolite, Readily 3D SA). To dissolve uncrosslinked resin, the printing container was incubated at 37 °C for 5 min before washing the constructs with pre‐warmed PBS. Hollow or perfusable constructs were subsequently perfused with fluorescently labeled material and cured in a UV‐box (BSL‐01, Opsytec Dr. Gröbel GmbH).

### FLight Printing

For Flight prints, 200 µL of the fluorescently labeled ColNB formulations were loaded into µ‐Slides (Ibidi) and thermal gelation was introduced at 4 °C in the dark. Flight projections were performed using a custom‐made device, with a light‐intensity of 60 mW cm^−2^ at 405 nm. The uncrosslinked resin was allowed to dissolve at 37 °C and was removed by washing with pre‐warmed PBS.

### Mechanical Testing

Unconfined compression tests were performed on a TA.XTplus Texture Analyzer (Stable Micro Systems) equipped with a 500 g load cell. Samples were placed between the two compression plates, and a pre‐load of 0.3 gram was applied to ensure that the samples were in full contact with the plates. After relaxation, samples were compressed to 15% strain at 0.01 mm s^−1^. Loading and unloading curves were recorded and the compressive modulus was calculated by linear fitting the first 3% of the stress–strain curve.

### Circular Dichroism (CD) Measurements

CD spectra of pristine collagen, gelatin, and norbornene functionalized collagen and gelatin solubilized at 0.1 mg mL^−1^ in 20 mmol HCL were recorded on a Jasco J‐815 CS spectrometer in the range of 190–280 nm. The solution was loaded into a high‐quality quartz cuvette (Hellma), and spectra with a bandwidth of 0.5 nm in continuous scanning mode were collected at 4 °C (*n* = 3). Collagen and ColNB subjected to iodixanol were purified via dialysis, lyophilized, and CD‐spectra were recorded after reconstitution. CD spectra were smoothed using a Savitzky–Golay filter with a window length of 15 and a third‐order polynomial function.

### Thermorheology

The temperature‐dependent rheological properties Collagen and ColNB were evaluated using an Anton Paar MCR 302e rheometer equipped with a 20 mm parallel plate geometry and a stainless‐steel floor. To prepare the collagen and ColNB solutions, polymers were dissolved in 20 mmol acetic and neutralized by the addition of 10x PBS and diluted with PBS to a final concentration of 4 mg mL^−1^. Additional samples were supplemented with 3% iodixanol, For the rheological measurements, 76 µL of the solution was applied onto the steel floor, the gap distance was set to 0.2 mm and shear measurements were performed at a shear rate of 2% and a frequency of 1 Hz, with acquisition intervals of 10 s (*n* = 2). To maintain sample integrity and prevent drying during testing, all measurements were conducted in the presence of a wet tissue paper in the chamber. In the first 5 min of the measurements, the temperature was maintained at 4 °C, then the temperature was increased to 25 °C for 10 min before a final increase to 37 °C for 30 min.

### Photorheology

Photorheology analyses were carried out on an Anton Paar MCR 302e equipped with a 20 mm parallel plate geometry and glass floor. Omnicure Series1000 lamp (Lumen Dynamics) was used in combination with sequential 400–500 nm and narrow 405 nm bandpass filters (Thorlabs). Photoresins were prepared as previously described. All procedures were performed in the dark. Oscillatory measurements were performed in triplicates at 37 °C using 76 µL of photoresins at 2% shear rate and 1 Hz frequency with 200 µm gap and 10 s acquisition interval. Measurements were left to proceed in the dark for 5 min before irradiating the sample with 405 nm light at 2 mW cm^−2^ intensity. To prevent the sample from drying, all tests were performed in the presence of a wet tissue paper in the chamber.

### Gelatin Release Experiments

UV‐cross‐linked hydrogel cylinders (3 mm diameter, 4 mm height) were used to characterize the release kinetics of Gel‐Rho. The gelatin‐containing bioresin was prepared as previously described, however instead of using 1% w/v gelatin, rhodamine‐labeled gelatin (Gel‐Rhod) was used. Resins were casted in PDMS molds and cross‐linked in an UV box (400 mJ cm^−^^2^, 405 nm). Individual hydrogel cylinders were placed in 1 mL of PBS at 37 °C. At designated time intervals, 500 µL of the supernatants were collected and replenished with fresh PBS. At the last timepoint, hydrogel cylinders were homogenized in the PBS solution. To quantify Gel‐Rho release, fluorescence measurements were conducted at an excitation wavelength of 560 nm and an emission wavelength of 510 nm. The percentage release at each timepoint was calculated by comparing the release at that specific timepoint to the cumulative release over all timepoints.

### Cell Culture and Seeding

C2C12 murine myoblasts and NIH 3T3 murine fibroblasts were obtained from ATCC and cultured in DMEM+GlutaMAX medium supplemented with 10% Fetal Bovine Serum (FBS) and 10 µg mL^−1^ gentamicin, herein referred to as growth medium (GM). All cells were cultured in tissue culture flasks in an incubator at 37 °C under a humidified atmosphere of 5% CO_2_. The cells were passaged at 80% confluency using 0.25% w/v trypsin and 0.05% w/v ethylenediaminetetraacetic acid. C2C12 differentiation was induced by incubating the C2C12 in DMEM supplemented with 2% v/v horse serum 1% v/v Insulin‐Transferrin‐Selenium (ITS+, Corning) and 10 µg mL^−1^ gentamicin, herein referred to as differentiation media (DM).

### Bioprinting and Cell Viability Assay

Bioresins were prepared as described above under aseptic conditions with the addition of + 10 µg mL^−1^ Antibiotic‐Antimycotic (Anti‐Anti). C2C12 or 3T3 were encapsulated at a concentration of 1 million cells mL^−1^. For cell‐tracker dye loading, cells were incubated in serum‐free DMEM containing either cell tracker red (Red CMPTX Dye, Invitrogen) or green (Cytopainter, abcam) for 1 h, cells were then washed three times in PBS and encapsulated. The fabrication procedure of cell‐laden tomographic or FLight constructs was like as described above. For multi‐cellular constructs, the first cell‐laden resin was loaded into the printing vials/cuvettes/µ‐slides and allowed to thermally solidify at 4 °C in the dark. Upon solidification, the resin containing the second cell type was loaded, the printing vessel sealed, and thermal gelation was induced. After the light projections were performed, the printing containers were incubated at 37 °C for 5 min to liquify the uncrosslinked portions. The harvested constructed was first washed thoroughly in PBS containing antibiotics and then in GM to remove all not encapsulated cells from the surface. The viability of encapsulated cells was assessed on day 1 after fabrication using DMEM supplemented with 1:2000 calcein‐AM (Invitrogen), 1:1000 Hoechst 33342 (Invitrogen), and 1:500 propidium iodide (PI, Fluka). For analysis, confocal laser scanning microscope (Fluoview 3000, Olympus) was used to acquire 100 µm stacks with 3 µm step distance (*n* = 3). Z‐stacks were reconstructed in Fiji Image J and analyzed.

### Immunofluorescence Staining

Cell‐laden hydrogel‐constructs were fixed in 4% v/v paraformaldehyde for 30 min at room‐temperature. Cells were permeabilized by incubation in 0.1% v/v Triton X100 in PBS for 15 min, samples were washed in PBS three times, and blocked with 5% w/v bovine serum albumin (BSA) in PBS for 1 h. Constructs were either incubated in primary anti myosin heavy chain antibody (MF‐20, DSHB, 1:20 in PBS + 1% v/v BSA), anti alpha‐actinin (sarcomeric) antibody (A7811, Sigma, 1:500 in PBS + 1% v/v BSA) or anti integrin‐b1 antibody (CD29 (TS2/16), Invitrogen, 1:100 in PBS + 1% w/v BSA) for 24 h under constant agitation. Constructs were washed three times in PBS and incubated in secondary antibody and dyes (Goat anti‐mouse AlexaFlour488, Invitrogen, 1:200 in PBS + 1% v/v BSA), Hoechst 33342 (H3570, Invitrogen, 1:1000 in PBS + 1% v/v BSA), phalloidin‐tetramethylrhodamine B isothiocyanate(P1951, Invitrogen, 1:1000 in PBS + 1% v/v BSA), CellMask (C10046, Invitrogen, 1:1000 in PBS + 1% w/v BSA) at 4 °C for 2 h. Before confocal imaging (Fluoview 3000, Olympus), samples were washed in PBS three times.

### Analysis of f‐Actin Alignment and Hydrogel Microarchitecture

For microstructure quantification of the FLight constructs, Fiji software was used.^[^ ^85^ ^]^ Confocal stacks were imported into the software, and sequential maximum intensity z‐projections with a thickness of 5 µm were created. Microchannels and microfilaments were characterized by using the plot profile function and diameters measured at the full width at half maximum (*n* = 3 hydrogels, dataset per hydrogel ≥ 60 measurements). For F‐actin alignment analysis, z‐projections of confocal images were used. Alignment was quantified using the Fiji OrientationJ plugin (available at http://bigwww.epfl.ch/demo/orientationj). Both for distribution and analysis identical tensor parameters (local window was set to 20 µm with gaussian gradient) were used. 3D porosity was calculated by dividing the volume occupied by microfilaments by the total volume based on 3D surface reconstruction using Imaris (Oxford Instruments, Ver. 10.2.0). For doing so, confocal stacks were converted into imaris compatible file format (Imaris File Converter software, Oxford Instruments, Ver. 10.2.0), next the surface was reconstructed using standard surface details and threshold was manually set to 285 and size exclusion filter of 1200 was applied to remove speckles from the reconstruction. The same analysis was carried out for 3 different hydrogel matrices per material and used to calculate 3D porosity.

### Light Sheet Microscopy

An axially scanned light sheet microscope (MesoSPIM, V4) was used to image the volumetrically printed constructs. The constructs were submerged in a quartz cuvette filled with MilliQ water, and the cuvette was mounted onto the MesoSPIM microscope stand. For imaging, a macro‐zoom system (Olympus MVX‐10) and 2x air objective (Olympus MVPLAPO1x) with adjustable zoom were used. Voltage adjustments using the electrically tunable lens (ETL) were performed for each run. Step size was 10–50 µm.

### Statistical Analysis

Statistical analysis was carried out in GraphPad Prism (v. 10.2.3). For comparisons between two groups, unpaired *t*‐tests were used. For comparisons involving more than two groups, one‐way ANOVA followed by appropriate post hoc tests (Tukey's or Holm–Šidák) was applied. Data are represented as mean ± SD, significance level alpha was set to 0.05, and statistical significance between groups was denoted as ^*^ *p* < 0.05, ^**^ *p* < 0.01, and ^***^ *p* < 0.005 and ^****^ *p* < 0.001, ns represents no significance.

## Conflict of Interest

The authors declare no conflict of interest.

## Supporting information



Supporting Information

Supplemental Video 1

## Data Availability

The data that support the findings of this study are openly available in ETH Research Collection at https://doi.org/10.3929/ethz‐b‐000712063, reference number 712063.
