# Supplementary material for: Rapid Deep Vat Printing Using Photoclickable Collagen‐Based Bioresins
Source: Adv Healthc Mater. 2025 Jul 4;14(28):2405105. doi: 10.1002/adhm.202405105 (PMC12581886; doi:10.1002/adhm.202405105)
Supplement: Supplementary file 1 — Supporting Information [file ADHM-14-0-s002.docx]

**Supplementary information**

**Rapid deep vat printing using photoclickable collagen-based bioresins**

Michael Winkelbauer^1^, Amelia Hasenauer^1^, Dominic Rütsche^1,2^, Hao Liu^1^, Jakub Janiak^1^, Michael Nguyen^3^, Karen L. Christman^3^, Marcy Zenobi-Wong^1^, Parth Chansoria^1*^

^1^ Department of Health Sciences and Technology, ETH Zürich, Switzerland

^2^ Department of Surgery, University Children's Hospital, Switzerland

^3^ Shu Chien-Gene Lay Department of Bioengineering, Sanford Stem Cell Institute, Sanford Consortium for Regenerative Medicine, University of California San Diego, La Jolla, California, USA

^#^Correspondence: [parth.chansoria@hest.ethz.ch](mailto:parth.chansoria@hest.ethz.ch)

**ORCIDs**

Michael Winkelbauer (<https://orcid.org/0009-0001-1035-7818>)

Amelia Hasenauer (<https://orcid.org/0000-0003-4512-6195>)

Dominic Rütsche (<https://orcid.org/0000-0001-6394-201X>)

Hao Liu (<https://orcid.org/0000-0002-8301-6870>)

Jakub Janiak (https://orcid.org/0009-0000-7162-6252)

Michael Nguyen (<https://orcid.org/0000-0003-1847-4557>)

Karen Christman (<https://orcid.org/0000-0002-6179-898X>)

Marcy Zenobi-Wong ([https://orcid.org/0000-0002-8522-9909](https://orcid.org/0000-0002-6107-6848))

Parth Chansoria (<https://orcid.org/0000-0002-6107-6848>)

This supplementary information consists of Figures S1 to S9. The corresponding methods have been provided in the main manuscript.


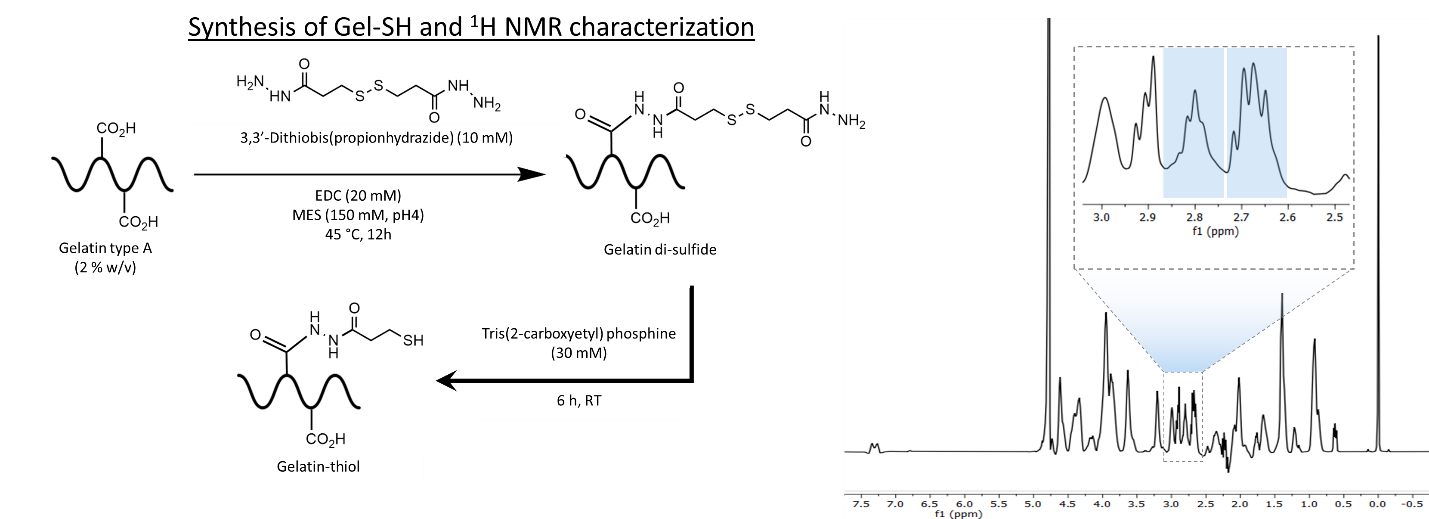


**Figure S1.** Synthesis of thiolated gelatin and the corresponding ^1^H NMR curve demonstrating methylene peaks (2.82 and 2.66 ppm). The integrals of the peaks in ColNB were compared to the methyl protons of the DSS internal standard (-0.05 to 0.05 ppm).^[1]^


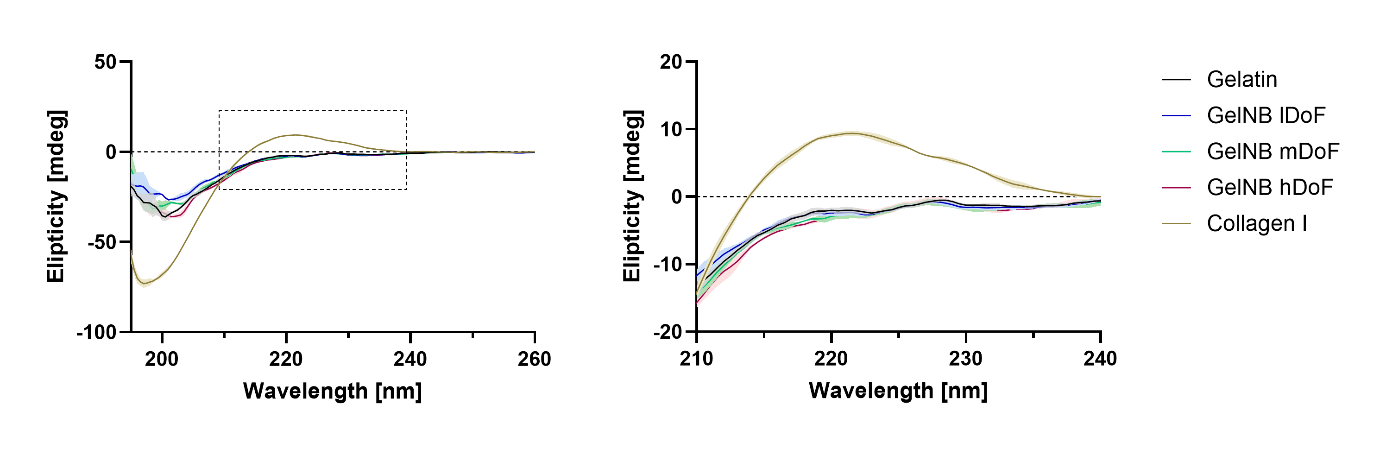


**Figure S2.** Circular dichroism (CD) spectra of pristine collagen, gelatin and norbornene-functionalized gelatin (GelNB) different degrees of functionalization (DoF). Synthesis of the GelNB at different DoFs: low (lDoF), medium (mDoF) or high (hDOF), was based on our prior work.^[2]^ All gelatins, whether functionalized or pristine, lacked the characteristic peak at 220 nm, indicating the absence triple-helical structure typically associated with native collagen.


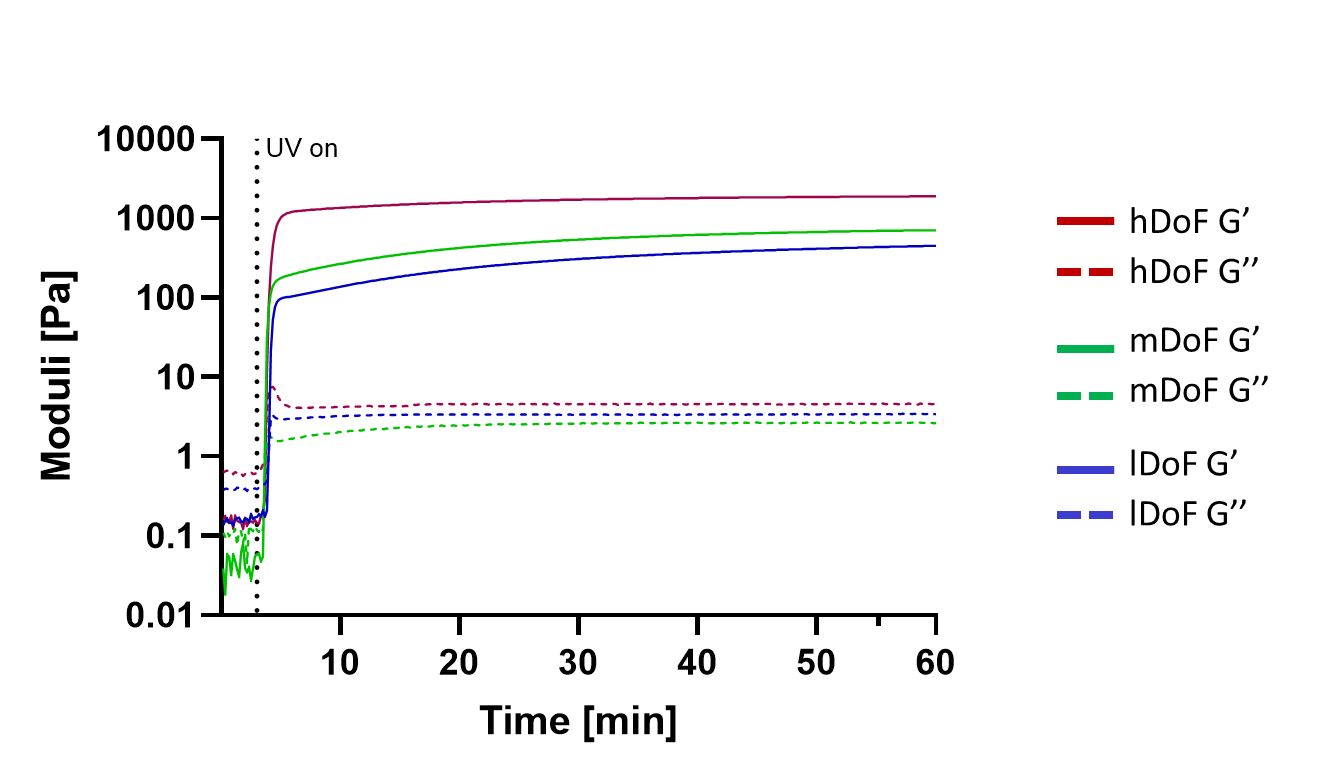


**Figure S3.** Photorheology for resin formulations containing norbornene-finctionalized collagen (ColNB) at different degree of functionalization (DoFs). The letters G’ and G’’ represent storage and loss moduli, respectively. The abbreviations hDoF, mDoF and lDoF represent high, medium and low degrees of functionalization, respectively. Each resin formulation contained 3 mg/mL ColNB and 25 mg/mL GelSH. 405 nm light was switched ON after 3 min.


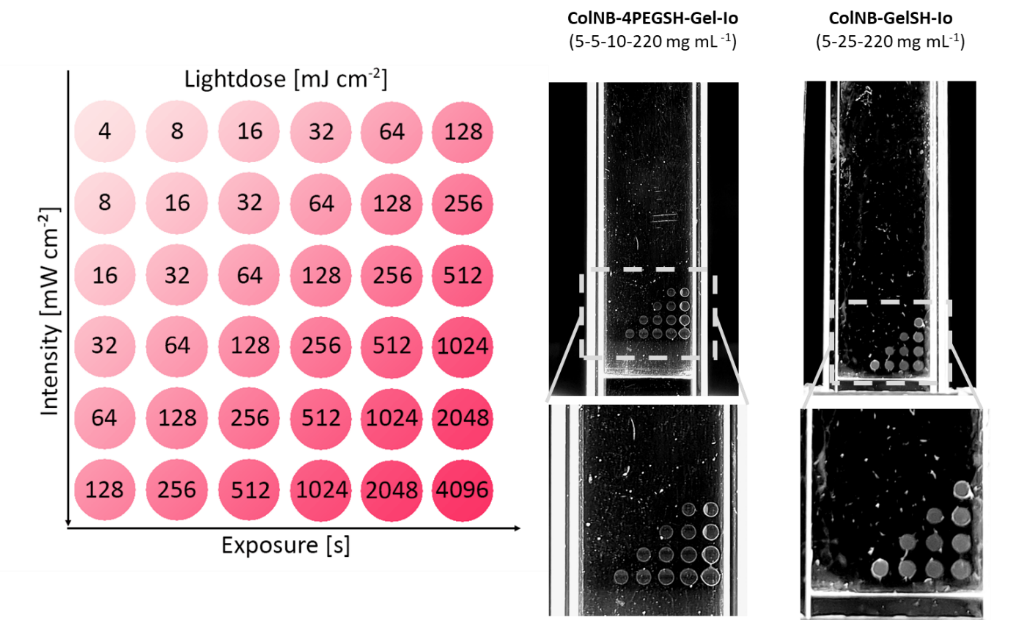


**Figure S4.** Dose tests of neutral collagen-based resin formulations. Optimal doses were 400 and 550 mJ cm­^-2^ for the formulations based on 4PEGSH and GelSH as crosslinkers, respectively.


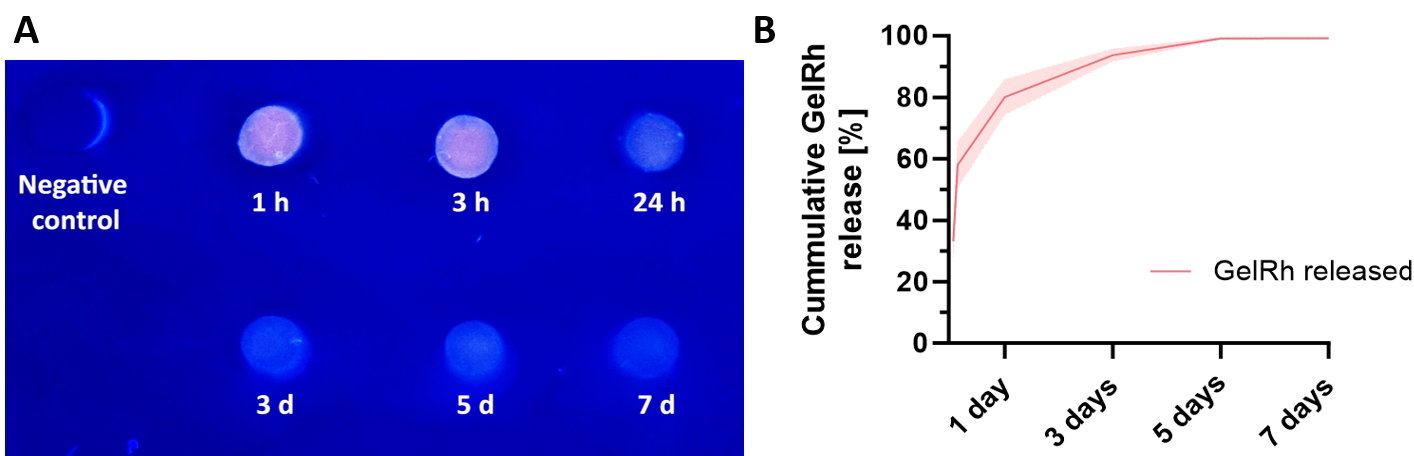


**Figure S5. A.** Fluorescent images of the constructs with the sacrificial gelatin labeled with Rhodamine (details in methods). The fluorescence of the constructs reduces as more gelatin is released. **B.** Release profile of the sacrificial gelatin from the constructs.

**
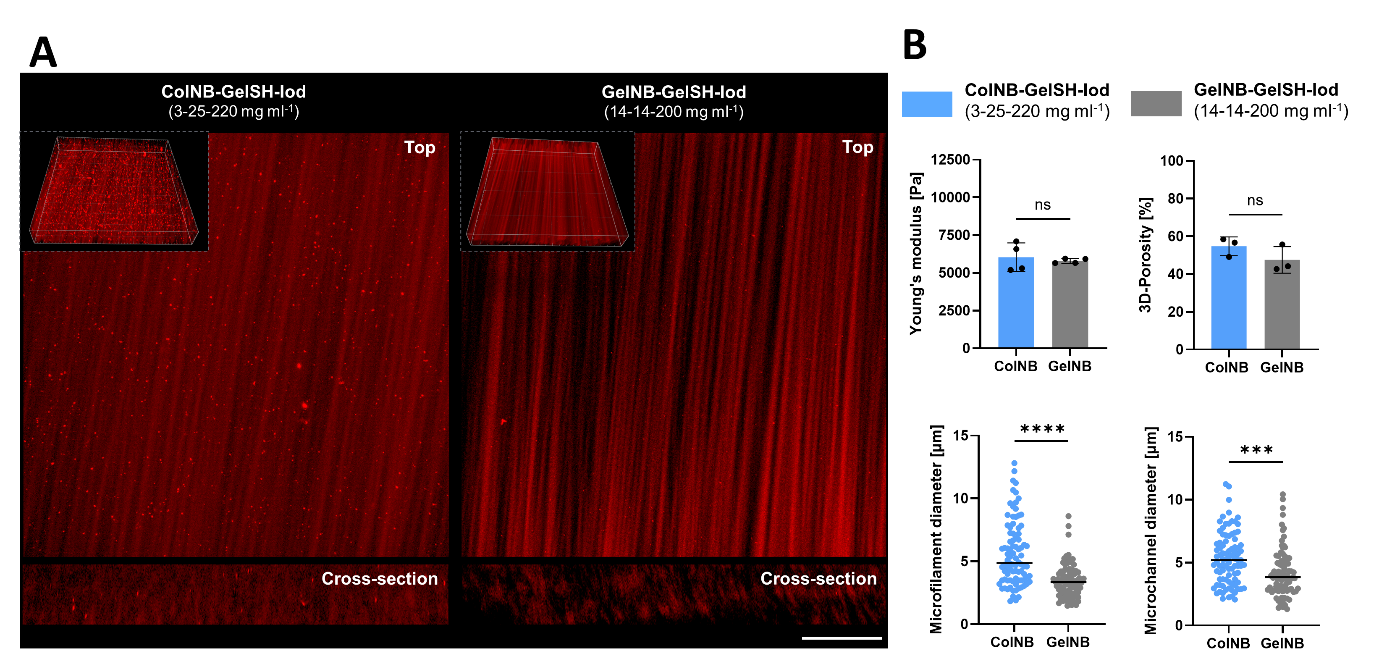
**

**Figure S6. Microarchitectural Characterization of ColNB and GelNB Photoclick Hydrogels with Matched Physical Properties.** **A.** Confocal image based 3D-reconstruction (isometric, top and front views) of rhodamine-labeled hydrogels showing microfilaments. **B.** Microarchitectural features and porosity were analyzed using resin formulations matched in polymer content (2.8 % w/v), refractive index (RI), and compressive modulus (~6 kPa). While overall porosity did not differ significantly between groups, both microfilament and microchannel diameters were significantly larger in ColNB-based hydrogels. Scale bar: 40 µm. Statistical significancy is denoted as follows: **** represents p<0.0001. and *** represents p<0.001.

**
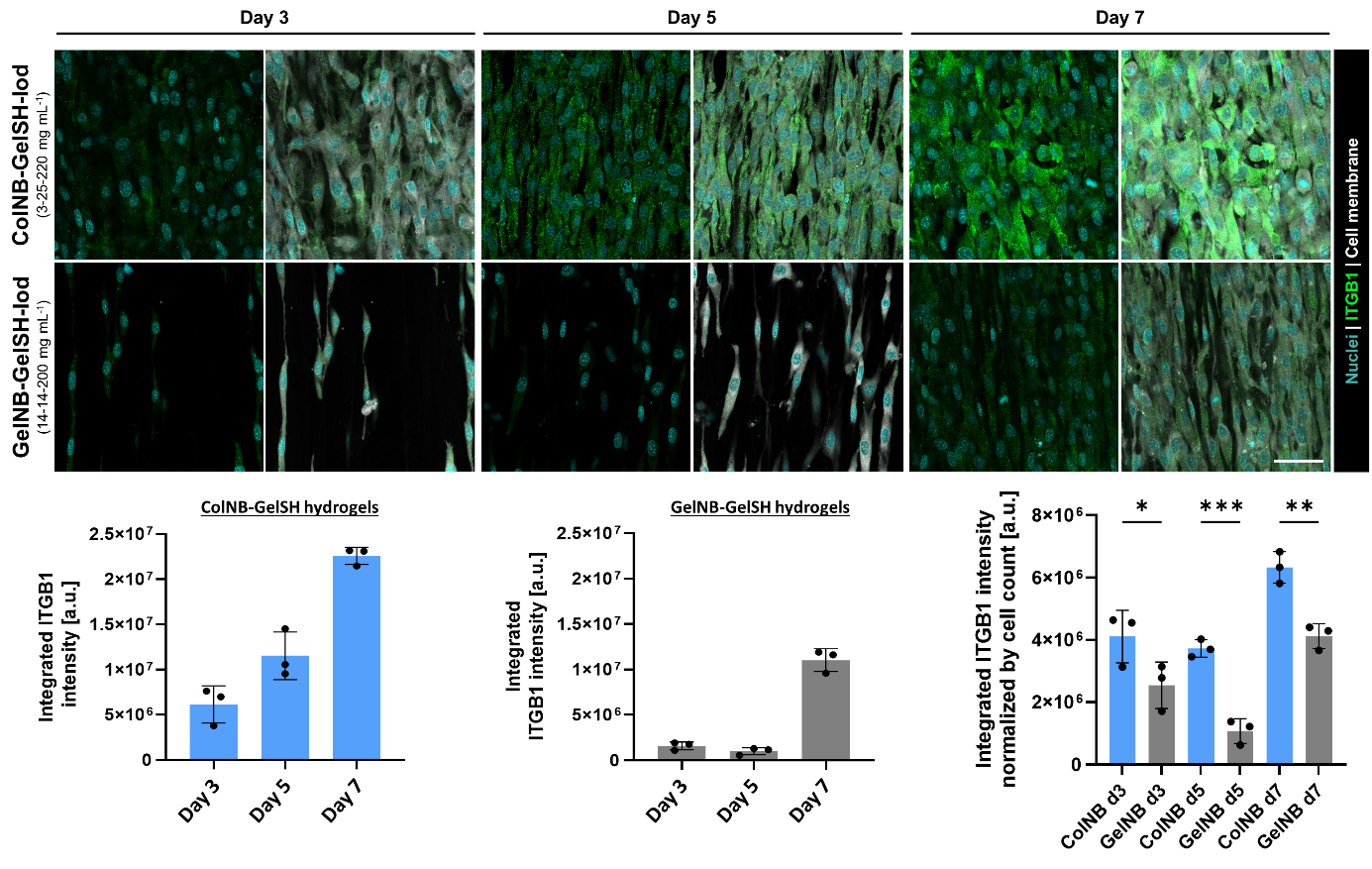
**

**Figure S7. Quantification of integrin β1 (ITGB1) expression in C2C12 myoblasts encapsulated in ColNB and GelNB–GelSH hydrogels, assessed over a 7-day culture period.** Immunofluorescent staining of C2C12 myoblasts encapsulated in GelNB-GelSH and ColNB-GelSH matrices. Integrated intensity of ITGB1 staining increases over time in both hydrogel conditions. Cells encapsulated in ColNB-based hydrogels consistently exhibited higher overall ITGB1 expression compared to those in GelNB at all timepoints. Upon normalization to cell count, ITGB1 expression in ColNB-based hydrogels remained elevated and followed a similar increasing trend. In contrast, cells within GelNB-based hydrogels showed a transient decrease in ITGB1 expression, followed by a gradual increase starting from day 5. Turquoise: Nuclei, green: integrin β1, gray: cell membrane. Scale bar: 50 µm. Statistical significancy is denoted as follows: *** represent p<0.001, ** represents p<0.01 and * represents p<0.05.


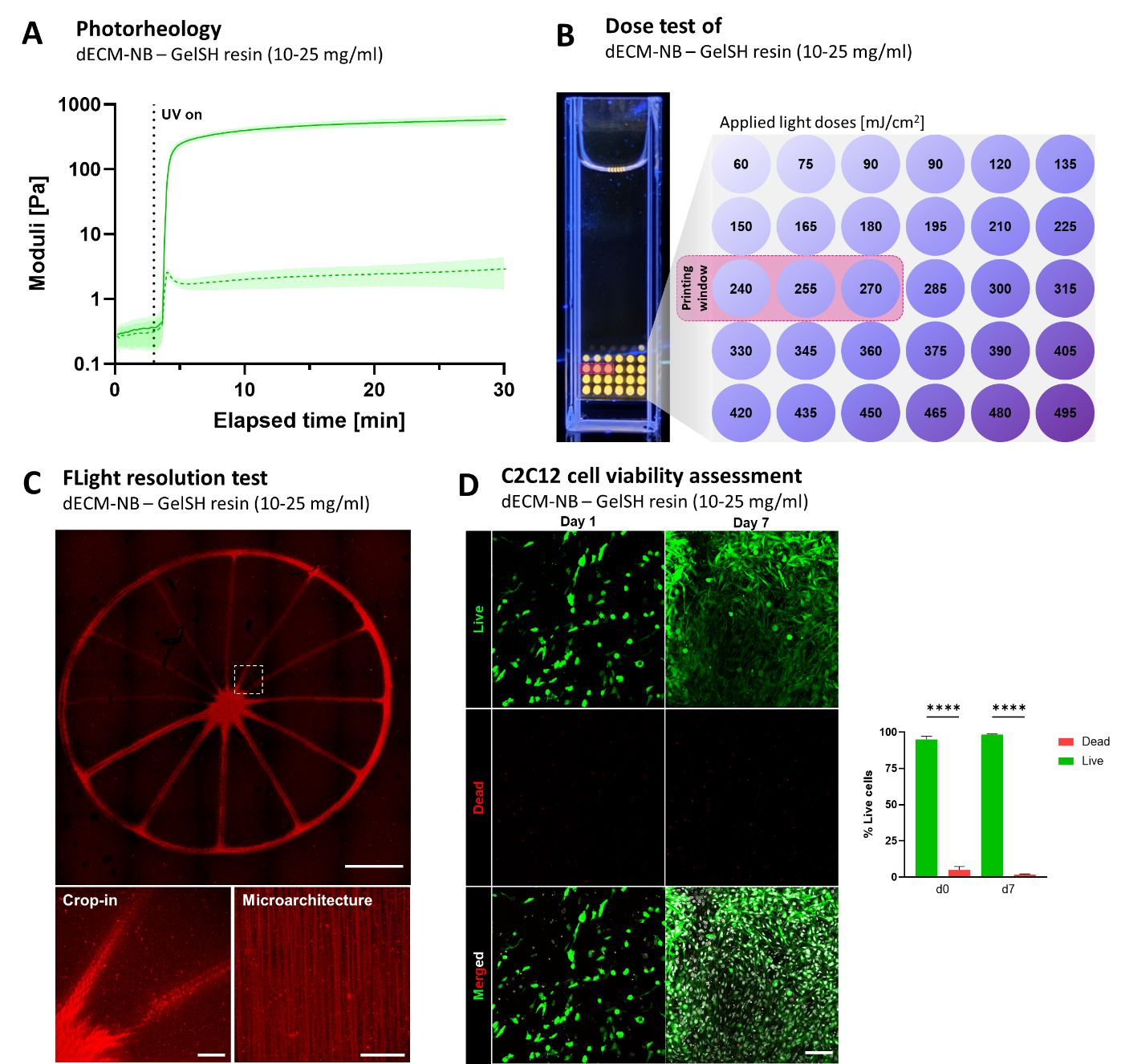


**Figure S8. Deep-vat printing of norbornene-functionalized decellularized extracellular matrix (dECM-NB).** Materials were sourced from the Christman group based on established methods.^[3,4]^ Norbornene functionalization approach was the same as that for Col I in other studies in this manuscript. **A.** Photorheological response of neutralized dECM-NB-GelSH reisn; solid and dotted line represent storage and loss moduli, respectively. **B.** Light-dose tests identified an optimal printing window between 240 and 270 mJ/cm². **C.** Crosslinked spoke wheel pattern to demonstrating high printing resolution and microarchitecture. Scale bars: Spoke wheel: 1 mm, crop-in: 100 µm, microarchitecture: 50 µm. **D.** Quantification of cell-viability of encapsulated C2C12 cells stained with propidium iodide (dead) and Calcein AM (live) after 1 and 7 days post- biofabrication. Data represented as mean ± SD (n=3), statistical significance was determined by t-test and is denoted as follows: **** refers to p<0.0001.

**
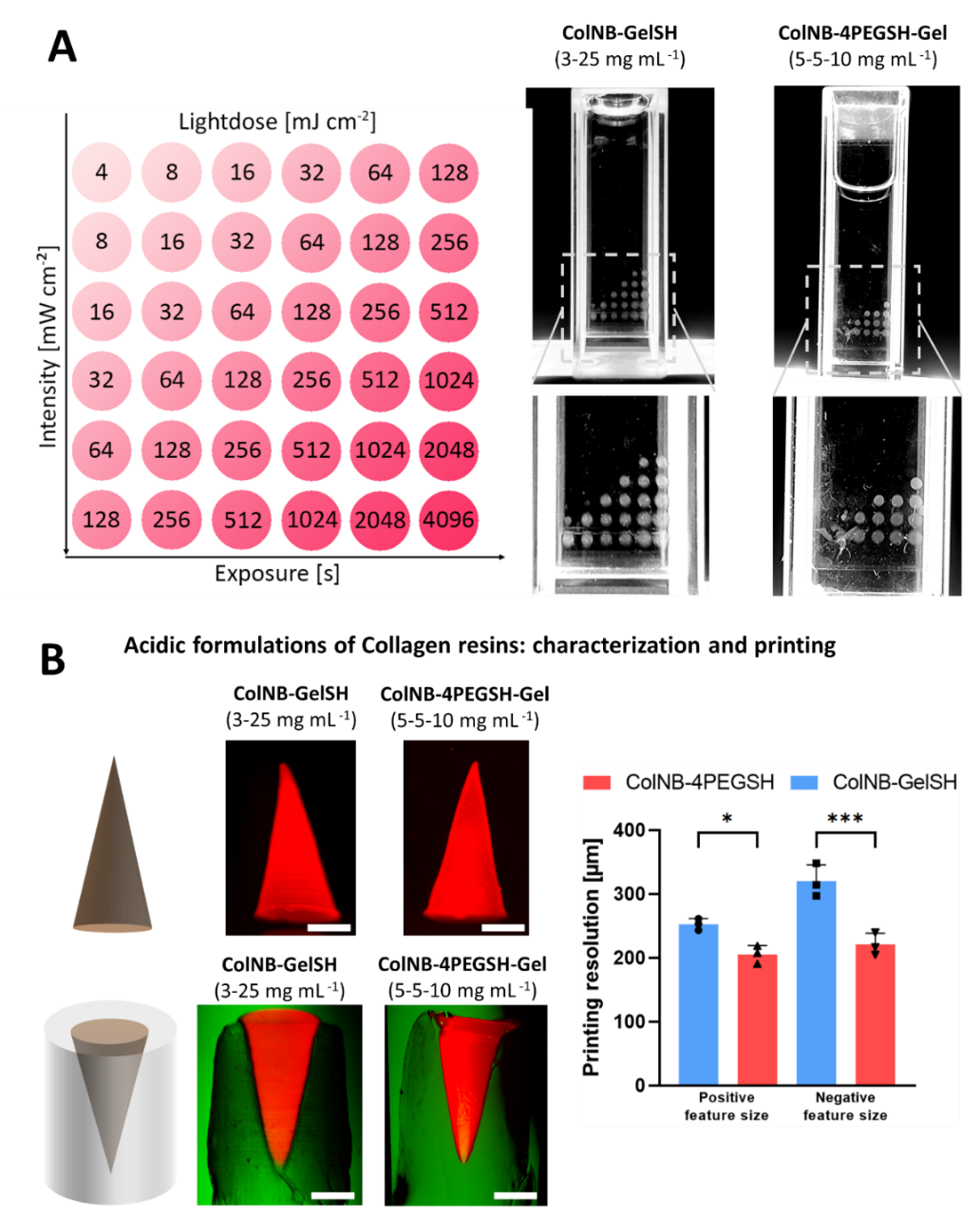
**

**Figure S9. Tomographic printing using acidic collagen formulations. A.** Dose tests of acidic collagen formulations (note that there is no Iodixanol present in the constructs). **B.** Tomographically printed positive and negative feature tests. Scale bars: 1000 µm.

**References**

[1] K. Guo, H. Wang, S. Li, H. Zhang, S. Li, H. Zhu, Z. Yang, L. Zhang, P. Chang, X. Zheng, *ACS Appl Mater Interfaces* **2021**, *13*, 7037.

[2] R. Rizzo, D. Ruetsche, H. Liu, M. Zenobi-Wong, *Advanced Materials* **2021**, *33*, 2102900.

[3] M. T. Spang, R. Middleton, M. Diaz, J. Hunter, J. Mesfin, A. Banka, H. Sullivan, R. Wang, T. S. Lazerson, S. Bhatia, et al., *Nat Biomed Eng* **2023**, *7*, 94.

[4] J. L. Ungerleider, T. D. Johnson, M. J. Hernandez, D. I. Elhag, R. L. Braden, M. Dzieciatkowska, K. G. Osborn, K. C. Hansen, E. Mahmud, K. L. Christman, *JACC Basic Transl Sci* **2016**, *1*, 32.
